# Chemokines in Type 1 Diabetes Mellitus

**DOI:** 10.3389/fimmu.2021.690082

**Published:** 2022-02-15

**Authors:** Xiongfeng Pan, Atipatsa C. Kaminga, Sanjay Kinra, Shi Wu Wen, Hongying Liu, Xinrui Tan, Aizhong Liu

**Affiliations:** ^1^ Department of Epidemiology and Health Statistics, Xiangya School of Public Health, Central South University, Changsha, China; ^2^ Department of Mathematics and Statistics, Mzuzu University, Mzuzu, Malawi; ^3^ Departmentof Non-Communicable Disease Epidemiology, London School of Hygiene and Tropical Medicine, London, United Kingdom; ^4^ Ottawa Hospital Research Institute (OMNI) Research Group, Ottawa Hospital Research Institute, Ottawa, ON, Canada; ^5^ Department of Obstetrics and Gynaecology, University of Ottawa Faculty of Medicine, Ottawa, ON, Canada; ^6^ School of Epidemiology and Public Health, University of Ottawa Faculty of Medicine, Ottawa, ON, Canada; ^7^ Department of Pediatrics, the Second Xiangya Hospital, Central South University, Changsha, China

**Keywords:** chemokines, type 1 diabetes mellitus, inflammatory, network meta-analysis, diabetes mellitus

## Abstract

**Background:**

Previous studies suggested that chemokines may play an important role in the formation and mediation of immune microenvironments of patients affected by Type 1 Diabetes Mellitus (T1DM). The aim of this study was to summarise available evidence on the associations of different chemokines with T1DM.

**Methods:**

Following PRISMA guidelines, we systematically searched in PubMed, Web of Science, Embase and Cochrane Library databases for studies on the associations of different chemokines with T1DM. The effect size of the associations were the standardized mean differences (SMDs) with corresponding 95% confidence intervals (CIs) of the chemokines concentrations, calculated as group differences between the T1DM patients and the controls. These were summarized using network meta-analysis, which was also used to rank the chemokines by surface under cumulative ranking curve (SUCRA) probabilities.

**Results:**

A total of 32 original studies on the association of different chemokines with T1DM were identified. Fifteen different chemokine nodes were compared between 15,683 T1DM patients and 15,128 controls, and 6 different chemokine receptor nodes were compared between 463 T1DM patients and 460 controls. Circulating samples (blood, serum, and plasma) showed that concentrations of CCL5 and CXCL1 were significantly higher in the T1DM patients than in the controls (SMD of 3.13 and 1.50, respectively). On the other hand, no significant difference in chemokine receptors between T1DM and controls was observed. SUCRA probabilities showed that circulating CCL5 had the highest rank in T1DM among all the chemokines investigated.

**Conclusion:**

The results suggest that circulating CCL5 and CXCL1 may be promising novel biomarkers of T1DM. Future research should attempt to replicate these findings in longitudinal studies and explore potential mechanisms underlying this association.

## Introduction

Type 1 diabetes mellitus (T1DM) affects about 11-22 million people worldwide and accounts for 90% of childhood diabetes (1, 2). The global incidence and prevalence of T1DM have markedly increased in recent years, as have their serious complications, resulting in a considerable health burden to individuals living with T1DM worldwide (3). Specifically, T1DM is an autoimmune disorder characterized by destruction of the pancreatic β cells (4). In this regard, dysfunction of the β cells leads to severely impaired, or absent insulin secretion, and hence patients need long-term insulin treatment to survive. However, the immunological trigger and the exact pathogenesis of T1DM remain unknown (5). It should be noted that long-term insulin treatment for T1DM has several other limitations (3). For example, although this treatment partly addresses the paradoxical and pathophysiological excess of glucagon, it is associated with weight gain; which adversely affect hypertension, cardiovascular disease, and atherosclerosis risk profile (6, 7). Therefore, studies have been conducted to explore new insulin adjunct therapies for T1DM. The findings of these studies showed an increasing body of evidence linking T1DM to pancreatic immune microenvironment (8–10).

Specifically, preclinical work has identified chemotactic cytokines (chemokines) as an important group of first inflammatory mediators, expressed after pancreatic early damage, which act to coordinate immune cells and attract them to the immune microenvironment of ongoing inflammation (11). A further study of the pancreatic immune microenvironment indicated a possible role of T helper 1 (Th1) cells in the progress of β cells destruction (8, 12). Moreover, the autoreactive memory T cells (CD4+ and CD8+ T cells) may lead to the progressive destruction of pancreatic β cells (13). In this process, chemokines mediate the rapid recruitment of activated Th1, neutrophils, macrophages, and natural killer (NK) cells, which increase the initial damage, hence mediating the killing of β cells and promoting further inflammation (10).

Considering that chemokines can be divided into four subfamilies, C, CC, CXC, and CX3C, according to their N-terminal cysteine motifs, the role of chemokines and their receptors in T1DM may be multifaceted (11). For example, previous studies indicated that the CCL4-CCR5 axis, CXCL10-CXCR3 axis, and CXCR1/2 pathway have the ability to predominantly attract the more aggressive Th1 T-cells, which increase the initial damage, hence mediating the killing of β cells and promote further inflammation (9, 10, 14). On the other hand, the CXCL12-CXCR4 axis and CXCL12-CXCR7 have the ability to sustain local immune-isolation, and can stimulate the regeneration, proliferation, and survival of β cells (15, 16). This evidence supports the hypothesis that there is a crosstalk between different chemokines that are involved in the *in situ* inflammatory responses, potentially contributing to both the initial destruction of β cells and the intensified progression to overt T1DM. Therefore, targeted immune intervention of critical chemokines may prevent the subsequent development of the autoimmunity. However, previous studies exploring chemokine biomarkers for T1DM have used multiple unstandardised names for chemokines and frequently found conflicting results, which have thus far not been summarized using meta-analysis. Therefore, this study aimed to quantitatively analyze the concentrations of different chemokines in T1DM, using high-quality meta-analysis techniques involving as many chemokine names as possible.

## Methods

### Search Strategy and Selection Criteria

This meta-analysis was conducted according to the Preferred Reporting Items for Systematic Reviews and Meta-Analyses (PRISMA) guidelines, and standard procedures provided in the Cochrane Handbook (17). In addition, the protocol for this meta-analysis was registered in the PROSPERO database (registration number: CRD42019148305 (https://www.crd.york.ac.uk/PROSPERO).

Following PRISMA guidelines, two authors (XP and AK) independently searched in the Web of Science, PubMed, Embase, and Cochrane Library, for relevant studies published not later than June 30, 2020. Additionally, the reference lists of selected studies were manually checked to find more relevant studies. Studies not written in English and grey literature were also included to avoid publication bias. Experienced librarians designed and adjusted a broad but highly structured search strategy. Specifically, professional and variant names were included to identify as many chemokines as possible according to the immunologists’ recommendations. Keywords for the search strategy also included various combinations of terms for T1DM, juvenile-onset diabetes mellitus and autoimmune diabetes. In addition, Boolean operators, truncation, and wildcards were implemented to allow for variant chemokines and T1DM names. The full search strategy is available in the **Appendix 1** of **Supplementary Information**.

### Study Selection

Studies were selected for data extraction based on the Population, Intervention/Exposure, Comparison, Outcome, and Study design (PICOS) framework. In this regards, the Population was patients with or without T1DM, Intervention/Exposure was patients with T1DM, Comparison was subjects without T1DM, Outcome was chemokines concentrations, and the Study design included longitudinal, cross-sectional, and case-control study designs. The inclusion criteria were: (1) studies measured concentrations of chemokines in patients with T1DM and controls, or these data could be obtained upon request from the corresponding authors; (2) studies reported methods for diagnosing T1DM; (3) controls did not meet diagnostic criteria for T1DM; and (4) study designs were longitudinal, cross-sectional, or case-control. The exclusion criteria were: (1) *in vitro* and non-human studies; (2) participants had a comorbid or additional metabolic disorders; (3) studies focused on stimulated levels of chemokines (these studies measured the consequences of pharmacological challenge as opposed to basal immune activity); and (4) conference papers, case reports, letters, or reviews were excluded.

### Data Extraction

Two reviewers (XP and AK) independently extracted the data using a custom data extraction template. First, Endnote (version x9.1) was used to remove duplicate data, and then the EpiData (version 3.0) was used to extract data. All data were stored in a custom data extraction template (Microsoft Excel 2019). For this study, the following characteristics of eligible studies were extracted: (1) surname of the first author and year of publication; (2) region of the research origination; (3) sample characteristics such as sample materials; (4) chemokines sample detection method; (5) characteristics of study subjects such as body mass index (BMI), race, duration of diabetes, age, sex, waist circumference (WC), systolic blood pressure (SBP), and diastolic blood pressure (DBP); (6) biochemical indicators such as fasting plasma glucose (FPG), C-reactive protein (CRP), low-density lipoprotein (LDL), high-density lipoprotein (HDL), triglycerides (TG), haemoglobin A1c (HbA1c) and creatinine; and (7) the mean and standard deviation [mean (± s.d.)] of chemokine concentrations of study subjects. Two independent reviewers sought missing data through request from corresponding authors *via* emails. When a request was not successful, alternative techniques were used for data extraction. For example, the Engauge Digitizer (version 4.1) was used for data extraction when data were presented only in graphical format. Any disagreements regarding data inclusion were settled by consensus involving the third reviewer (AL). Finally, the Newcastle–Ottawa Quality Assessment Scale (NOS) was used to assess the risk of bias and the quality of the eligible studies as recommended by the Cochrane Collaboration. Moreover, studies that scored greater than or equal to the median quality score (≧̸4) were considered having low risk of bias (18, 19).

### Statistical Analysis

The package meta of R software (version 3.5.2) was used for meta-analysis, and Stata (version 15.0) was used for network meta-analysis (20, 21). The effect size was estimated as standardized mean difference (SMD) with corresponding 95% confidence interval (CI), using Cohen’s d. Given racial and assay methods heterogeneity, when measuring chemokines, the random-effects model was used to summarize the effect size (22–24). The random-effects model includes both within-study and between-studies variation in the assessment of the uncertainty of results (25, 26). This meta-analysis method is more conservative and is chosen if significant heterogeneity is expected. Statistical heterogeneity between studies was assessed using the Cochrane’s Q test (Chi-squared test) (27). Further, the *I*² statistic was calculated to quantify this statistical heterogeneity (28). The *I*² ranges from 0% to 100%, with 100% indicating maximal heterogeneity and 0% no heterogeneity (29). If significant heterogeneity exists, it suggests variability in the study characteristics. The likely sources of heterogeneity in this study included BMI, race, HbA1c, age, sex, and duration of diabetes. Therefore, whenever data were adequate, these variables were investigated by subgroup analysis to explore sources of heterogeneity. Sensitivity analyses were performed to explore the impact of an individual study on the estimated effect size, SMD. This was accomplished by omitting each study at a time (19). Lastly, the funnel plot and the Egger’s test were used to assess publication bias when at least 10 studies per comparison were available (30).

Network meta-analysis was conducted by the Markov chain Monte Carlo method in Stata (version 15.0), using the self-programmed Stata routines and network commands, to compare the effects of multiple correction chemokines. The random effects model was evaluated before conducting the network meta-analysis. Thus, if the random effects model satisfied some conditions, network meta-analysis was performed in a frequentist framework, with consistency assumption (31). The first 10,000 iterations were discarded, and additional 50,000 iterations were executed (20). Moreover, vague priors were used in the network meta-analysis. A common heterogeneity value across all comparisons was assumed, and the average residual deviation was used to estimate the Goodness-of-fit. Standardized mean differences (SMDs) along with their 95% CI were presented for continuous data in the networks. Meanwhile, for each of the chemokines, the surface under cumulative ranking curve (SUCRA) was used to estimate the relative rankings of the various chemokines (32). Thus, chemokines with higher SUCRA values were considered the most potent chemokines. The value, SUCRA=0, indicated the worst significant difference, whereas SUCRA=1 indicated the best significant difference. A p value (two-sided) <0.05 indicated statistically significant results in all the statistical tests.

## Results

### Literature Search

The utilization of the systematic search of electronic databases and manual searching of references yielded a total of 3,732 studies, of which 903 were from the PubMed, 1,228 from the Embase, 768 from the Web of Science, and 833 from the Cochrane library. After duplicates were removed, 3,368 abstracts were reviewed and this resulted in the exclusion of 3,113 studies. Following a full review of 255 studies, 32 met the inclusion criteria for this systematic review and meta-analysis (**Appendix 2**).

### Characteristics of Eligible Studies

Characteristics of included studies are shown in **Table 1** and **Appendix 3**. These studies were published between 2001 and 2019. Nine different CC chemokine nodes were compared between 10,013 T1DM patients and 9,713 controls, while 6 different CXC chemokine nodes were compared between 5,670 T1DM patients and 5,415 controls. Further, 6 different chemokine receptor nodes were compared between 463 T1DM patients and 460 controls. As regards sample materials, 21 studies examined chemokines from blood samples, 9 from serum samples, and 2 from plasma samples. Five studies were conducted in the United States of America; 3 in Germany; 2 in each of the countries, Canada, China, Greece, Iran, Italy, and UK; and 1 in each of the countries, Belgium, Brazil, Egypt, Estonia, Finland, Hungary, India, Japan, Norway, Serbia, Sweden, and Turkey. To determine chemokines, 21 studies used enzyme linked immunosorbent assay (ELISA), 6 used Luminex, 3 used Multiplex, and 2 used radioimmunoassay (RIA). The real-time polymerase chain reaction (RT-PCR) was used to detect the mRNA expression of chemokine receptor. The NOS scores of study quality assessment varied between 5 and 8, with ten studies classified as moderate quality and 22 as high quality.

**Table 1:** Characteristics of included studies.

Study		Material	Country	Male (%)	NOS	BMI	Mean Age	Detection method	Duration of diabetes
**Abke et al. 2006**	(33)	Blood	Germany	0(0.0%)	8	22.3(19.5-31)	36.5(18.0-46.0)	ELISA	13.5 (7–34) years
**Antonelli et al. 2008**	(34)	Serum	Italy	49(51%)	7	17.1 ± 3.2	8.2 ± 3.7	ELISA	NR
**Berg et al. 2010**	(35)	Blood	Sweden	47(56.6%)	7	NR	9.5(1.6-16.4)	ELISA	NR
**Cetinkalp et al. 2015**	(36)	Blood	Turkey	9(36.0%)	6	25.2(3.6)	33.3 ± 9.2	ELISA	NR
**Chatzigeorgiou et al. 2010**	(37)	Plasma	Greece	17(38.6%)	8	20.48 ± 1.03	11.5 ± 0.7	ELISA	50.76 ± 11.61 months
**Dakovic et al. 2013**	(38)	Serum	Serbia	9(45.0%)	7	NR	12.8 ± 3.9	ELISA	5.78 ± 3.45 years
**Ellina et al. 2012**	(39)	Plasma	Greece	13(43.3%)	7	20.65 ± 0.95	11.5 ± 0.6	ELISA	57.53 ± 19.61 (0–184) months
**Erbağci et al. 2001**	(40)	Blood	USA	NR	5	NR	NR	ELISA	NR
**Gabbay et al. 2012**	(41)	Serum	Brazil	35(42%)	8	18.6 ± 2.6	13.0 ± 5.0	ELISA	3 months
**Giulietti et al. 2007**	(42)	Blood	Belgium	6(46.15%)	8	31.3(24.5-40.0)	62.0(48.0-81.0)	ELISA	8.8 years (3-29 years)
**Guan et al. 2011**	(43)	Blood	China	0(0.0%)	5	NR	NR	ELISA	NR
**Hakimizadeh et al. 2013**	(44)	Blood	Iran	209(51.0%)	7	NR	30.0 ± 5.0	ELISA	10 ± 4 years
**Heier et al. 2015**	(45)	Blood	Norway	156(49.68%)	8	20.8(3.9)	13.7(2.8)	ELISA	5.5 ± 3.4 years
**Huang et al. 2012**	(46)	Serum	China	155(53.45%)	7	NR	26.4(0.9-61)	RIA	1.4 months (0-12 months)
**Ismail et al. 2016**	(47)	Blood	Egypt	13(33.3%)	8	23.51 ± 4.14	14.6 ± 2.6	ELISA	5.36 ± 3.32 years
**Jamali et al. 2013**	(48)	Blood	Iran	55 (51%)	7	NR	45.0 ± 9.5	ELISA	10 ± 4 years
**Lappin et al. 2015**	(49)	Blood	UK	10(35.7%)	7	NR	35.0 ± 10.0	ELISA	NR
**Lohmann et al. 2002**	(50)	Blood	Germany	NR	5	NR	9.9 ± 4.1	ELISA/FLA	3.2 ± 1.2 years
**Melo et al. 2016**	(51)	Serum	Canada	10(27.8%)	8	23.6 ± 4.9	14.5 ± 1.4	Luminex	8.0 ± 3.4 years
**Nicolett et al. 2002**	(52)	Blood	India	0(0.0%)	6	25.7	27.6	ELISA	NR
**Nieminen et al. 2012**	(53)	Blood	Finland	NR	5	NR	7.3 ± 1.4	Luminex/qPCR	NR
**Pellegrini et al. 2017**	(54)	Blood	Italy	10(52.63%)	7	NR	34.0(6.0-65.0)	ELISA/RT-PCR	20 (0–37) months
**Pham et al. 2012**	(55)	Serum	Germany	62(68.88%)	8	26.1(22.8-29.2)	43.2(35.5-53.3)	Multiplex	1.0 (0.2-1.6) years
**Powell et al. 2018**	(56)	Blood	UK	5(50%)	6	NR	33.4(23.0-44.0)	ELISA/FLA	NR
**Purohit et al. 2015**	(57)	Blood	USA	266(47.5%)	8	NR	24.6 ± 16.5	Multiplex	10.7 ± 9.8 years
**Rosa et al. 2008**	(58)	Blood	USA	13(61.9%)	7	65.4 ± 5.4	13.4 ± 0.3	Luminex	NR
**Sickle et al. 2009**	(59)	Blood	USA	8(47.05%)	8	23.5 ± 4.1	15.9 ± 1.8	RIA	6.7 ± 2.2 years
**Sochet et al. 2017**	(60)	Serum	Canada	25(49%)	7	NR	14.8(10.9-16.8)	Luminex	6.7 (2.0-13.9) years
**Vorobjova et al. 2019**	(61)	Serum	Estonia	6(66.66%)	6	NR	8.5(4.0-13.1)	Multiplex	NR
**Wolkow et al. 2008**	(62)	Blood	USA	NR	5	NR	32.0(25.0-37.0)	Luminex	NR
**Yamamura et al. 2019**	(63)	Serum	Japan	NR	5	23.5 ± 4.1	53.5 ± 15.0	ELISA	12.1 ± 9.3 years
**Zóka et al. 2015**	(64)	Blood	Hungary	16(47.05%)	8	23.61(22.13-25.08)	32.7(29.4-35.9)	Luminex/FLA	14.6 years

NR, not report; BMI, Body Mass Index; RT-PCR, Real-time polymerase chain reaction; qPCR, Quantitative real-time polymerase chain reaction; FLA, Fluorescein-labeled antibody surface protein measurements; RIA, Radioimmunoassay; ELISA, Enzyme linked immunosorbent assay; USA, United States of America; UK, United Kingdom.

**Appendix 4** summarizes the correspondence between the classification of chemokines and their receptors. The XCL1 and XCL2 belong to the C subfamily of chemokines; the CCL1, CCL2, CCL3, …, CCL26, CCL27, and CCL28 belong to the CC subfamily of chemokines; the CXCL1, CXCL2, CXCL3, …, CXCL14, CXCL15, and CXCL16 belong to the CXC subfamily of chemokines; and the CX3CL1 belongs to the CX3C subfamily of chemokines. Moreover, the XCR1 belongs to the XCR subfamily of chemokine receptors; the CCR1, CCR2, CCR3, …, CCR8, CCR9, and CCR10 belong to the CCR subfamily of chemokine receptors; and the CXCR1, CXCR2, CXCR3, CXCR4, CXCR5, CXCR6, CXCR7, and CX3CR belong to the CXCR subfamily of chemokine receptors. **Appendix 5** summarizes the distribution of immune cells corresponding to different chemokine receptor types (e.g., Th1 cells, Th17 cells, CD4+T cells, CD8+T cells, Treg cells, basophils, dendric cells, monocytes, macrophages, and NK cells).

### Main Outcomes

**Figure 1:**
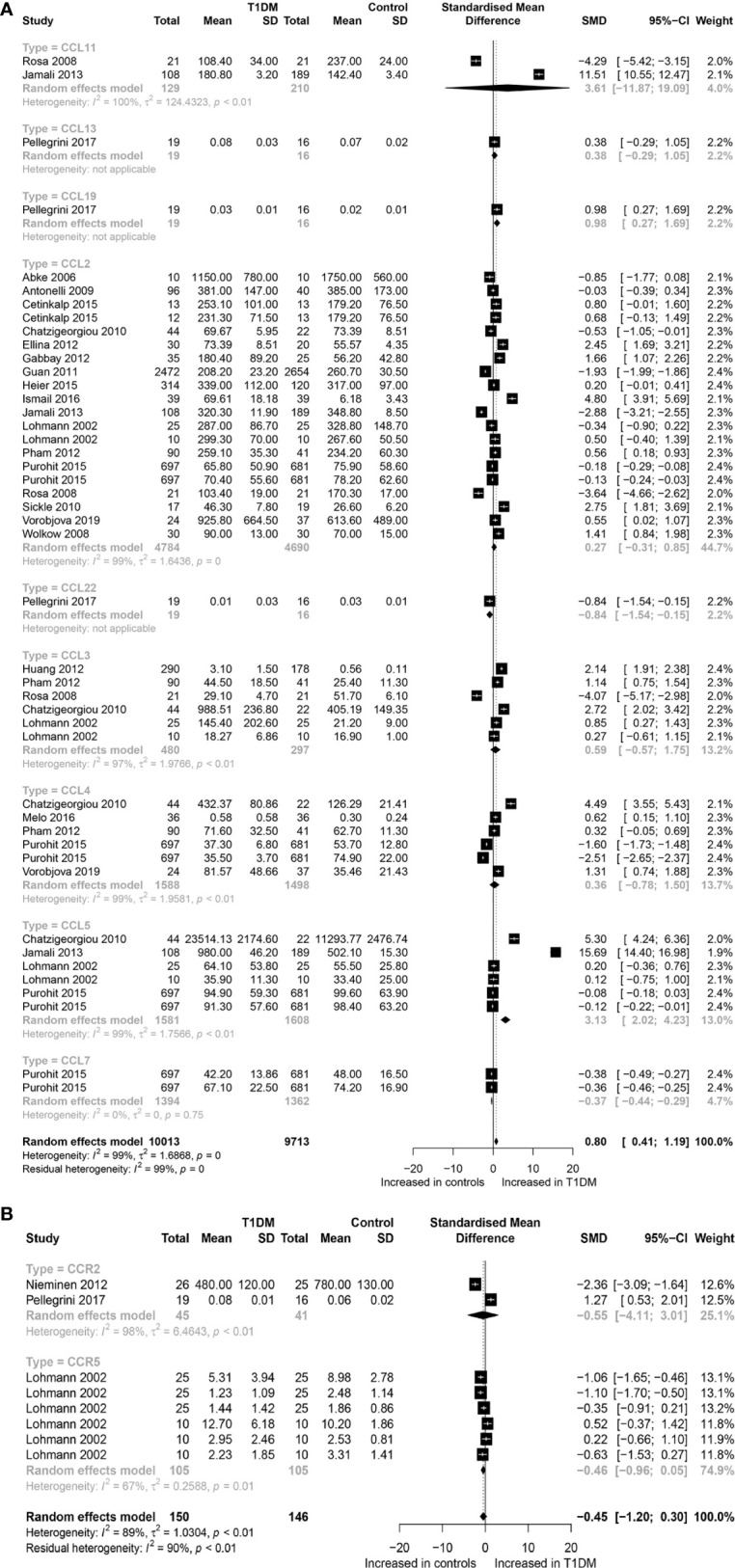
Forest plot of CC chemokines **(A)** and CCR **(B)** between T1DM patients and controls. Study effect sizes of chemokines differences between T1DM and controls. Each data marker represents a study, and the size of the data marker is proportional to the total number of individuals in that study. The summary effect size for each chemokines is denoted by a diamond. T1DM, Type 1 diabetes mellitus; CCR, CC chemokines receptor; SMD, standardized mean difference.

**Figure 2:**
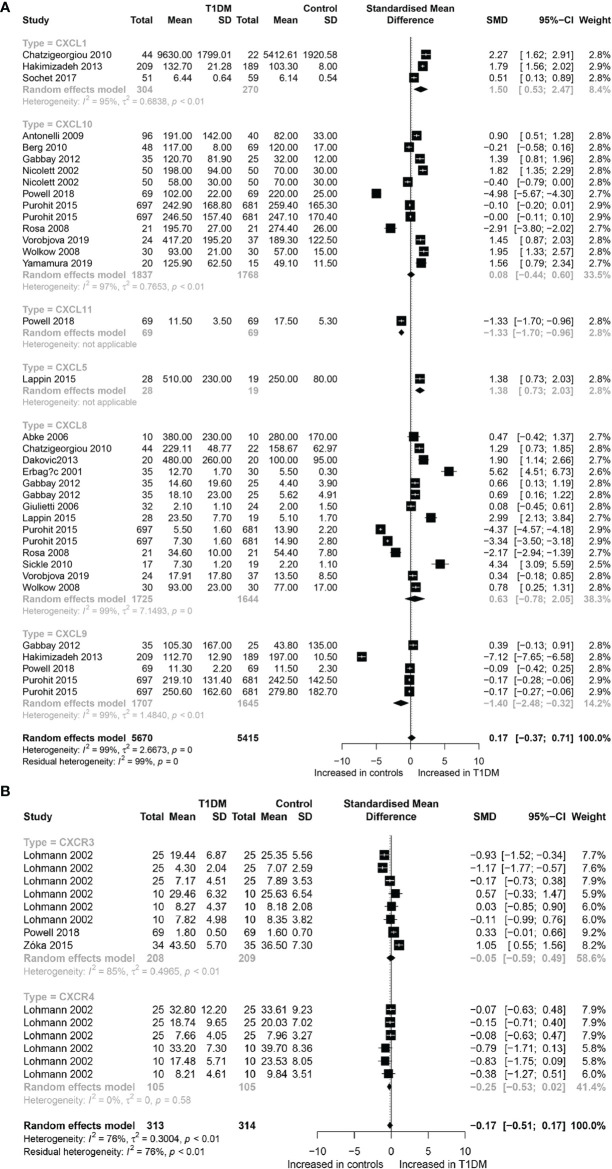
Forest plot of CXC chemokines **(A)** and CXCR **(B)** between T1DM patients and controls. Study effect sizes of chemokines differences between T1DM and controls. Each data marker represents a study, and the size of the data marker is proportional to the total number of individuals in that study. The summary effect size for each chemokines is denoted by a diamond; T1DM, Type 1 diabetes mellitus; CXCR, CXC chemokines receptor; SMD, standardized mean difference.

A total of 19,726 participants (10,013 T1DM cases and 9,713 controls) were included for analyzing the effect of CC chemokines on T1DM. Concentrations of CC chemokines in the circulating samples (blood, serum, and plasma) were significantly higher in the T1DM patients than in the controls (SMD=0.80; 95% CI: 0.41 to 1.19; **Figure 1**). Concentration of circulating CCL5 was significantly higher in the T1DM patients than in the controls (SMD=3.13; 95% CI: 2.02 to 4.23; **Figure 1**). Nevertheless, significant heterogeneity was observed across the included studies (*I*²=99%). However, there was a significant decrease in the concentrations of circulating CCL7 in the T1DM patients than in the controls (SMD=-0.37; 95% CI: -0.44 to -0.49; **Figure 1**). Evidence of heterogeneity across the included studies was not present (*I*²=0%). There were no significant differences in the concentrations of other CC chemokines between the T1DM and control subjects. Furthermore, a total of 11,085 participants (5,670 T1DM cases and 5,415 controls) were included in the analysis of the effect of CXC chemokines on T1DM. Concentrations of circulating CXCL1 were significantly higher in the T1DM patients than in the controls (SMD=1.50; 95% CI: 0.53 to 2.47; **Figure 2**). However, significant heterogeneity was observed across the included studies (*I*²=95%). Also, there was a significant decrease in the concentrations of circulating CXCL9 in the T1DM patients than in the controls (SMD=-1.40; 95% CI: -2.48 to -0.32; **Figure 2**). Similarly, significant heterogeneity was observed across the included studies (*I*²=99%). There were no significant differences in the concentrations of other CXC chemokines between the T1DM and control subjects. As regards the effect of chemokine receptors on T1DM, a total of 923 participants (463 T1DM cases and 460 controls) were included in the analysis. There were no significant differences in the concentrations of any chemokine receptors between the T1DM patients and controls. Sensitivity analyses demonstrated that eliminating any single study had little effect on the overall SMD of the concentrations of chemokines or chemokine receptors between the T1DM patients and controls. Additionally, there was no obvious asymmetry in the funnel plot (**Appendix 6**), and the Egger’s test was not significant (p=0.1139).

### Subgroup Analysis Outcomes

#### Subgroup Analysis for CC Chemokines

Subgroup analysis by race revealed that, among the Asian subjects, the concentrations of CC chemokines differed significantly between the T1DM patients and controls (SMD=3.79; 95% CI: 1.38 to 6.20; **Table 2**), but not among the Caucasian subjects. Similarly, subgroup analysis by sex revealed that, among the female subjects, the concentrations of CC chemokines differed significantly between the T1DM patients and controls (SMD=0.68; 95% CI: 0.23 to 1.14; **Table 2**), but not among the male subjects. Furthermore, subgroup analysis by BMI found a significant difference in the concentrations of CC chemokines, between the T1DM patients and controls, among the study subjects with a BMI of at most 23.9 (SMD=1.55; 95% CI: 0.67 to 2.43; **Table 2**), but not among those with a BMI > 23.9. Also, subgroup analysis by glycated haemoglobin status demonstrated a significant difference in the concentrations of CC chemokines, between the T1DM patients and controls, among the study subjects with HbA1c higher than 8% (SMD=2.09; 95% CI: 1.01 to 3.18; **Table 2**), but not among the study subjects with HbA1c lower than or equal to 8%.

**Table 2:** Subgroup analysis of CC and CXC chemokine between T1DM participants and controls.

Subgroup		SMD	95%-CI		Heterogeneity		
					Q	τ²	I²
**CC chemokine**		0.80	0.41	1.19	5932.26	1.69	99.30%
**Material**	Blood	0.44	0.00	0.87	4578.68	1.42	99.30%
	Plasma	2.86	0.73	4.99	159.53	5.71	97.50%
	Serum	0.92	0.34	1.50	150.67	0.73	94.70%
**Race**	White	0.23	-0.08	0.54	2190.10	0.82	98.40%
	Asian	3.79	1.38	6.20	2808.05	11.90	99.80%
**Sex**	Female	0.68	0.23	1.14	3432.09	1.10	99.40%
	Male	1.15	-0.21	2.51	1862.46	7.53	99.20%
	NR	0.44	-0.04	0.93	21.81	0.30	72.50%
**Age**	Adult	0.96	0.46	1.46	3374.99	1.37	99.40%
	Children/adolescents	0.73	0.07	1.40	586.60	2.37	96.40%
	NR	-1.93	-1.99	-1.86	0.00	–	–
**BMI**	>23.9	-0.05	-1.24	1.13	321.87	3.48	97.20%
	≤23.9	1.55	0.67	2.43	263.23	1.89	96.60%
	NR	0.81	0.31	1.31	4853.82	1.53	99.50%
**Duration**	>60	1.31	0.74	1.87	2946.16	1.24	99.50%
	≤60	1.14	0.58	1.70	366.91	1.44	95.10%
	NR	-0.87	-2.00	0.26	538.37	3.19	98.30%
**HbA1c**	>8%	2.09	1.01	3.18	1790.57	6.58	98.80%
	≤8%	-0.75	-2.64	1.14	238.97	7.20	97.10%
	NR	-0.10	-0.64	0.44	3467.49	1.11	99.60%
**CXC chemokine**		0.17	-0.37	0.71	4935.27	2.67	99.30%
**Material**	Blood	-0.54	-1.22	0.14	4426.50	2.67	99.50%
	Plasma	3.00	0.97	5.03	46.63	3.06	95.70%
	Serum	0.83	0.56	1.11	21.00	0.11	61.90%
**Race**	White	0.27	-0.27	0.81	3864.43	2.27	99.20%
	Asian	-0.47	-3.39	2.45	955.66	11.03	99.60%
**Sex**	Female	0.33	-0.34	1.01	3320.49	2.19	99.50%
	Male	-0.76	-2.10	0.57	1344.46	5.92	99.10%
	NR	2.42	0.82	4.01	60.37	2.49	95.00%
**Age**	Adult	-0.48	-1.20	0.25	4349.28	2.69	99.60%
	Children/adolescents	0.69	0.13	1.26	223.86	1.14	93.70%
	NR	5.62	4.51	6.73	0.00	–	–
**BMI**	>23.9	0.09	-1.40	1.58	178.79	3.31	97.20%
	≤23.9	1.06	0.69	1.42	29.24	0.22	72.60%
	NR	-0.19	-0.90	0.53	4459.54	2.72	99.60%
**Duration**	>60	-0.38	-1.26	0.49	4007.36	2.71	99.70%
	≤60	1.10	0.58	1.61	25.54	0.33	80.40%
	NR	0.30	-0.53	1.14	640.18	2.79	97.70%
**HbA1c**	>8%	0.60	-0.44	1.64	976.13	4.16	98.60%
	≤8%	0.67	-1.40	2.73	176.98	6.47	97.20%
	NR	-0.44	-1.20	0.32	3328.68	2.20	99.60%

Subgroup analyses are performed to compare the concentration of chemokines and chemokines receptors between the T1DM and the controls. Heterogeneity was quantified using I^2^ and its significance was tested using the Q statistics. T1DM, Type-1diabetes mellitus, NR, not report; SMD, standardized mean difference.

#### Subgroup Analysis for CXC Chemokine

Subgroup analysis by age group demonstrated a significant increase in the concentrations of CXC chemokines, between the T1DM patients and controls, among the children/adolescents (SMD=0.69; 95% CI: 0.13 to 1.26; **Table 2**), but not among the adults. Likewise, subgroup analysis by sample type revealed a significant difference in the concentrations of CXC chemokines, between the T1DM patients and controls, when the plasma samples were used (SMD=3.00; 95% CI: 0.97 to 5.03; **Table 2**); and when the serum samples were used (SMD=0.83; 95% CI: 0.56 to 1.11; **Table 2**); but this was not the case when the whole blood samples were used. Also, subgroup analysis by BMI revealed a significant difference in the concentrations of CXC chemokines, between the T1DM patients and controls, among the study participants with a BMI <= 23.9 (SMD=1.06; 95% CI: 0.69 to 1.42; **Table 2**), but not among the study participants with a BMI > 23.9. Similarly, subgroup analysis demonstrated a significant difference in the concentrations of CXC chemokines, between the T1DM patients and controls, when the duration of T1DM was < 60 months (SMD=1.10; 95% CI: 0.58 to 1.61; **Table 2**), but not when the duration of T1DM was >= 60 months.

**Figure 3:**
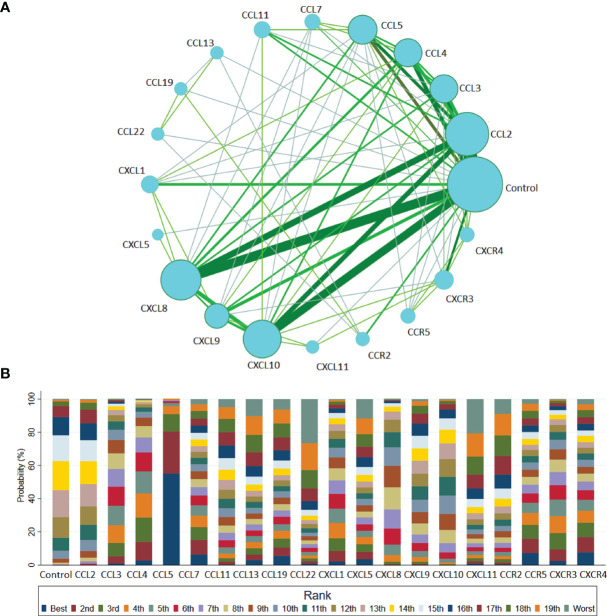
Network meta-analysis of chemokines comparisons for T1DM **(A)** and rank probability of chemokines in T1DM group for response rate in the network analysis **(B)**. The width of lines is proportional to the number of studies comparing every pair of chemokines. The size of each circle is proportional to the sample size (i.e., number of participants). **(A)** This plot shows the relative cumulative probabilities for each of the chemokines in the network. SUCRA values are presented in the legend. SUCRA, surface under cumulative ranking curve **(B)**.

Among the 32 studies, 20 nodes involving different chemokines, between the T1DM patients and controls, were included in the network analysis. **Figure 3A** presents the network graph. Additionally, **Figure 3B** presents the cumulative rank probability plot. This plot shows the relative cumulative probabilities for the chemokines in the network. Therefore, the CCL5 chemokine had the highest SUCRA probability (95.8%) and was ranked the highest in T1DM, followed by circulating CCL4, CCL3, CXCR3, CXCL1, CXCR4, CXCL8, CCL7, CCR5, CXCL10, CCL19, CXCL9, CXCL5, CCL11, CCL13, CCL2, CCR2, CXCL11 and CCL22.

## Discussion

To our knowledge, this is the most comprehensive systematic review of the role of chemokine systems in the pathophysiological process of T1DM. Chemokines and their receptors are expressed throughout in both the patients with T1DM and healthy subjects, under both inflammatory and physiological conditions. Our study indicates that a number of these chemokines (i.e., CCL5, CCL7, CXCL1, and CXCL9) discriminate between those with and without T1DM.

Inflammation appears to be involved in the interplay among chemokines and progression of T1DM, as this has been shown in other several pathological conditions (65–68). Moreover, recent reviews suggested that the chemokines, CXCL10, CXCL9, and CXCL11, are implicated in the pathogenesis of autoimmune diseases such as autoimmune thyroiditis, T1DM, Graves disease, Thyroid eye disease, and Addison’s disease (69–72). Also, evidence indicated that CCL2 and CXCL10 chemokines, modulated by cytokines and PPARγ agonist, play an important role in Graves’ ophthalmopathy (73). Specifically, PPARγ agonist activation plays an inhibitory role on CXCL10, but stimulates the release of CCL2. Moreover, a recent systematic review and meta-analysis suggested that the chemokines, CCL3, CCL4, CCL5, CCL20, CXCL8 and CXCL11, are implicated in the pathogenesis of non-alcoholic fatty liver disease, post-traumatic stress disorder, and also different types of cancers (74–77).

**Figure 4:**
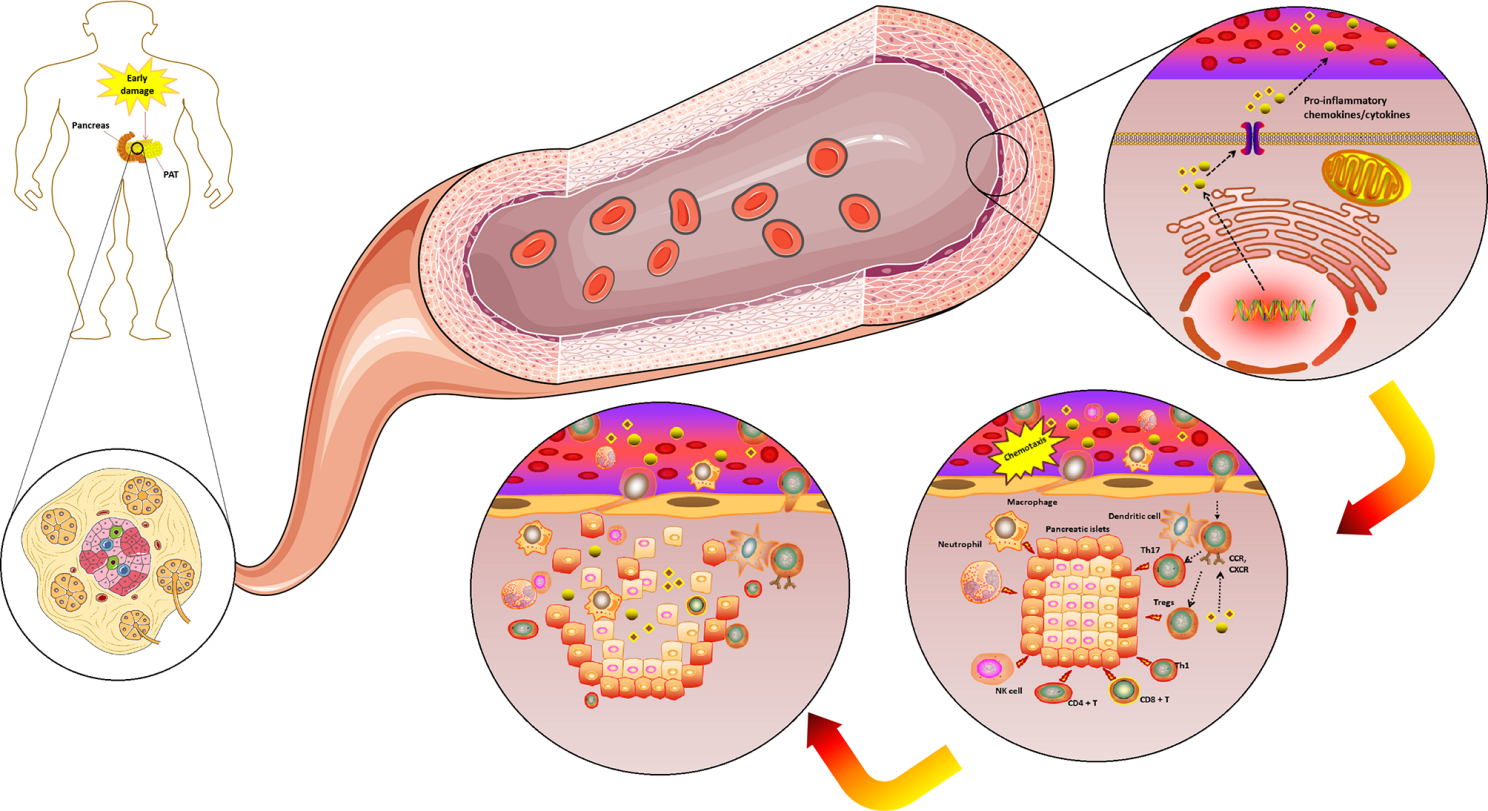
The complicated chemokines and their receptors network in the microenvironment of T1DM. The chemokine system plays a variety of roles in the T1DM microenvironment. Pancreatic islets and PAT are exposed to an early damage and start to secrete numerous pro-inflammatory chemokines/cytokines. The effective chemokines and their receptors can also cause a variety of immune cells to enter the pancreatic islets and PAT site to play the role of immune attack. T1DM progression is characterized by a massive and progressive secretion of pro-inflammatory chemokines/cytokines caused by pancreatic β cell death. Due to this process, various immune cell types (i.e., neutrophils, macrophages, NK cell, dendritic cell and specifically T cells) are recruited in the pancreatic tissue. These immune cells further release more innate inflammatory cytokines, which contribute to a rapid increase β cell death. T1DM, Type 1 diabetes mellitus; PAT, peri-pancreatic adipose tissue; CCR, CC chemokines receptor; CXCR, CXC chemokines receptor; NK, Natural killer; Tregs, Regulatory T cells. (Drawn by AK).

Furthermore, T1DM is believed to be an immunocytes-mediated autoimmune disease, whose microenvironment is influenced by the chemokines system in a variety of ways. For example, pancreatic islets and peri-pancreatic adipose tissue (PAT) are exposed to early damage and start to secrete numerous pro-inflammatory chemokines. Also, the effective chemokines and their receptors can cause a variety of immune cells to enter into the pancreatic islets and PAT site, where they simulate an immune attack. Noteworthy, T1DM progression is characterized by a massive and progressive secretion of pro-inflammatory chemokines caused by selectively destruction of insulin-producing β cells in the pancreas. Therefore, due to this process, various immune cell types (i.e., neutrophils, macrophages, NK cell, dendritic cell and specifically T cells) are recruited in the pancreatic tissue. These immune cells further release more innate inflammatory cytokines, which contribute to the rapid increase of β cell death. Thus, the preceding evidence support the hypothesis of a cross talk between different chemokines that are involved in the progressive auto-reactive immune response, and potentially contribute to both the initial pancreatic damage and the intense progression to overt T1DM (**Figure 4**). Specifically, this hypothesis is further strengthened by the evidence that, in humans, increased CCL5 was observed in the blood of newly diagnosed T1DM patients (50). These data indicated that CCL5 may be an important novel biomarker of T1DM (48). Moreover, CCL5 is identified as the dominant chemokine (in an inducible nitric oxide synthase-dependent but not NF-kappa B-dependent fashion) expressed *in vivo* in the islet inflammatory microenvironment of prediabetic animals and T1DM patients (78). Nevertheless, more indications toward the role of CCL5 during the pathogenesis of T1DM have been derived from cell experiments and animal models. For example, in the nonobese-diabetic (NOD) mouse, it was demonstrated that islet-specific Th1 but not helper T-cell 2 (Th2)-type cells produced CCL5 and promoted rapid induction of autoimmune diabetes (79). Another study that analyzed pancreas lysates found a massive increase in CCL5 levels at around ten weeks of age, which was found to remain high until at the age of at least 20 weeks (80). These elevated levels of CCL5 most likely reflected the degree of infiltration of pancreatic islets by autoaggressive T-cells, which are capable of producing CCL5. It was reported that CCR5 expression was detected in the islet lymphocytes and spleen of NOD mice; and it is also well known that CCL5 binds to the chemokine receptors, CCR5, which may contribute to the T1DM development (81).

Interestingly, results of another study indicated that proinflammatory cytokine produced from PATs not only significantly up-regulates CCL5 expression in islets, but also has direct cytotoxic effects on pancreatic islets. Besides, CCL5 is responsible for the recruitment of immune cells, which induces persistent inflammation in pancreases through the acquisition of Th1 and Th17 effector T cell subsets at the onset of T1DM (82). This evidence is consistent with our findings from both meta-analysis and network meta-analysis, which suggested that CCL5 may play a central role in the pathogenesis of T1DM.

However, our results also showed that the levels of circulating CCL7 decreased in T1DM. This is not consistent with a previous animal study, which found that CCL2, CCL5 and CCL7 were significantly expressed in NOD mice with T1DM (83). This contradiction may be due to the differences between animals and humans, or lack of data from human studies. Therefore, the results of CCL7 concentrations in T1DM should be interpreted cautiously. Further investigation is warranted on how CCL7 directly contributes to T1DM progression.

We also found that measured circulating CXCL1 levels were significantly higher in the T1DM patients than in the healthy subjects. This trend was also observed in animal studies (37, 44). Using the microarray approach, a study found a prominent expression of CXCL1, CXCL5 and CXCL9 in the NOD mice with T1DM (83). Generally, murine β cells can secrete large amounts of chemokines such as CXCL1; and cytokines such as IL-1α, and IL-6 that can, by themselves, improve leucocyte recruitment, and hence play a role in the pathophysiology of T1DM (84). A high affinity receptor of CXCL1, designated CXCR2, is physiologically expressed on the cell surface of different leukocyte subgroups (i.e., endothelial cells, basophils, NK cells, neutrophils, monocytes, and macrophages). Thus, reactive oxygen species (ROS), environmental stresses, or inflammatory signals such as IL-1 and TNF-α may induce CXCL1 expression and the activation of CXCR2 pathway (85). During inflammation, different leukocyte subgroups cause extravasation from the blood vessel, and recruitment to inflamed tissue is tightly regulated by the chemokine system. Furthermore, we speculate that the CXCL1-CXCR2 pathway may be involved in neutrophil activation and increased vascular permeability, hence different leukocyte subgroups may further be activated and migrate towards the damaged tissue (86). Since CXCL1 is thought to play a crucial part in the T1DM pathogenesis, alongside different leukocyte subgroup recruitment, anti-inflammatory strategies need to be further investigated.

Additionally, prediabetes animal studies suggested that CXCL10 is one of the major chemokines expressed *in vivo* in the islet environment (78, 87). That is, in the islet of prediabetes animal, β cells can regulate the autoimmune response by producing CXCL10, under the action of inflammatory factors such as IFN-γ and TNF-α. Thus, CXCL10 can induce the migration of Th1 lymphocytes into the islet and secrete more IFN-γ and TNF-α, then further stimulate β cells to produce chemokines, hence initiating and perpetuating the autoimmune cascade (88). Mechanistically, another study found that in the pancreases of autoimmune diabetic mice, the expression of CXCL10 chemokine, which attracts plasma cell like dendritic cells (pDCs), was significantly increased, and this resulted in a significant increase in the number of pDCs in the islet microenvironment (89). Moreover, pDCs play a role in promoting diabetes (89). In addition, findings of a previous study suggested that blocking of CXCL10 can impede the expansion of peripheral Ag-specific T cells and hinder their migration to the islet microenvironment (90). Furthermore, the CXCL10 blockade aborts Ag-specific injury of β cells in the islet microenvironment and abrogates T1DM (91). In this regard, it was shown that, after the treatment of CXCL10 DNA vaccination (pCAGGS-CXCL10), the spontaneous diabetic mice could induce the production of anti-CXCL10 Ab and inhibit the occurrence of spontaneous diabetes *in vivo* (92, 93). Specifically, the CXCL10 DNA vaccination resulted in the proliferation of islet β cells in the spontaneous diabetic mice model, and helped to maintain the quality of pancreatic β cells (93). All the above evidence indicates that the CXCL10 plays a key role in the development of T1DM in animal models. However, our study did not find any significant difference in the concentrations of CXCL10 between the T1DM and control groups. This may suggest that the role of CXCL10 in T1DM vary between species. Future studies should verify the role of CXCL10 in T1DM in larger sample sizes.

Considering the redundant nature of the chemokine system signaling, the most efficient intervention tactic should directly target the chemokine receptors. Although various approaches for blocking the chemokine receptor pathways are available, such as small molecules, peptide-derived or neutralizing antibodies, and inhibitors, they are in the preclinical stage and more randomized clinical trials are needed to prove their effectiveness in the future (94).

*In vitro* chemotaxis assays confirmed that lymphocytes infiltrating human islets, once stimulated by inflammatory cytokines (TNF-α and IFN-γ), are able to modulate the autoimmune response through the production of CXCL9. That is, CXCL9 can induce the migration of Th1 lymphocytes into the islet and secrete more TNF-α and IFN-γ, in this way initiating and perpetuating the autoimmune cascade (70). Moreover, it was shown that CXCL9 could promote the chemotaxis of activated NK cells and T cells through selective high affinity binding to CXCR3 (95). Interestingly, a study that evaluated the effects of genetic variability of CXCL9 and its dependence on the risk of T1DM in the German population, did not find an association of the CXCL9 polymorphisms with T1DM (96). However, our results indicated decreased levels of CXCL9 in the T1DM patients. Considering these opposing findings, our results on the association of CXCL9 with T1DM should be interpreted with caution. Further studies are needed to reveal why CXCL9 was reduced in T1DM.

The results of subgroup analysis indicated that significant differences in circulating CC chemokine concentrations, between the T1DM patients and controls, depended on type of race, or sex. This is in agreement with the findings of previous studies, which suggested that there are race and sex differences in the function of chemokine genes (97, 98). Therefore, we speculate that race and sex differences in the pathophysiological process of T1DM may be an important factor in stratification and individualized treatment of T1DM patients. Further, subgroup analysis showed that the circulating concentration of CC chemokines was significantly different between the T1DM patients and controls in participants with BMI < 23.9, or HbA1c > 8%. This may be due to the disorder of glucose and lipid metabolism in obese T1DM patients, which further destroys the homeostasis of the immune system and chemokines. Also, subgroup analysis revealed that materials from different sample sources significantly affected CXC chemokine concentrations in T1DM patients in that these concentrations differed significantly between patients with T1DM and controls, when plasma or serum samples were used, but this was not the case when whole blood samples were used. These results suggest that whole blood may not be a stable sample source for chemokines. Therefore, we recommend that serum or plasma samples should be used to detect CXC chemokines in future studies. Furthermore, subgroup analysis revealed that age and duration of diabetes significantly affected circulating CXC chemokine concentrations in T1DM patients. That is, unlike among adults and patients with T1DM for less than 60 months, there was a significant difference in circulating CXC chemokine concentrations between the T1DM patients and controls among children/adolescents and those with T1DM for at least 60 months. These results are consistent with the findings from age related inflammation kinetics studies, which suggested that patients gradually lose the ability to control excessive inflammatory response with the increase in age and duration of T1DM (99, 100). Also, when compared with healthy controls, a characteristic feature of patients with T1DM is that they have self-reactive T cells with a memory phenotype (13). These autoreactive memory T cells are likely to be long-lived and strongly responsive to antigenic stimulation with less dependence on costimulation for activation and clonal expansion, and hence may be the source of differences in circulating chemokine concentrations between the patients with T1DM and the controls (8, 13).

### Strengths and Limitations

This study has some strengths and limitations. First, we analyzed a large number of chemokines due to the use of a comprehensive search strategy during literature search. However, some chemokines with stronger glycosaminoglycan (GAG)-binding effect have concentration levels below the detectable limit in the blood circulation. Therefore, more sensitive methods should be used to detect chemokines in circulating blood in the future. For example, future studies can use Luminex liquid phase protein chip technology and Novogene high-quality sequencing technology, to detect multiple chemokines as comprehensively as possible in a single study. Second, confounding factors such as smoking, drinking, and blood pressure, were not measured or adjusted for in the included original studies, which might have affected the stability of the chemokines concentrations. Moreover, subgroup analysis suggested that circulating samples (blood, serum, and plasma) could be potential sources of heterogeneity on the association between chemokines and T1DM. Future studies should consider these confounding factors and address them during data analysis. Third, the original studies did not report data related to the onset of symptoms of T1DM. Thus, we categorized the time of disease duration as >=5 years, or < 5 years, a time when the pancreases of patients with T1DM have already been destroyed, and when the chemokine profile is probably quite different from that at the time of pancreatic injury. Therefore, if the purpose of future research is to analyze the potential role of chemokines and their receptors in the pathogenesis of T1DM, then the research should be conducted earlier during the onset of symptoms of the disease. However, in our study, the circulating concentrations of chemokines in the long-term course of the disease could be used as potential biomarkers. Fourth, the results of this study only describe the relationship between chemokines and T1DM status, which can provide important hypotheses for studies on causal relationships. The causal association between chemokines and pathogenesis, diagnosis and treatment of T1DM was not explored in this study due to the fact that all the original studies were case-control studies, and lacked data on the pancreas donors and onset of symptoms of T1DM. In addition, at the time this study was conducted, there was no cohort study investigating the causal association between chemokines and T1DM. Therefore, the findings of this study cannot describe any causal relationship between chemokines and T1DM. We recommend that population-based cohort studies should be conducted in future to determine the role of chemokines in the pathogenesis of T1DM, or the possible causal/dose-response relationship between T1DM and chemokines, and these studies should be performed on samples obtained not only from circulating samples but also pancreas donors. Further, our study did not find an association between CXCL10 and T1DM, although previous studies found significant associations between CXCL10 and diabetes in animal models. Finally, despite some limitations, the findings of our study have helped to clarify the intricacies of previous results, and suggest that chemokines have a significant correlation with the state of T1DM; hence can most likely serve as biomarkers for T1DM.

## Conclusions

The results of this study suggest that circulating CCL5 and CXCL1 are altered in T1DM patients. In addition, circulating CCL7, CXCL9 and CXCL10 may be underlying biomarkers for T1DM. Future studies are needed to ascertain causal associations between chemokines and T1DM.

## Data Availability Statement

The raw data supporting the conclusions of this article will be made available by the authors, without undue reservation.

## Author Contributions

XP and AL contributed to the study design, while XP and AK contributed to the data collection. Statistical analyses and interpretation of results were performed by AL, XT, and AK, whereas XP, XT, SK, HL, AL, and SW drafted the manuscript and edited the language. All the authors participated in the critical revisions, and approved the final version of the manuscript.

## Funding

The research is financially supported by Hunan Provincial Key Laboratory of Clinical Epidemiology and the Hunan Provincial Key Research and Development Program (2018SK2065), China.

## Conflict of Interest

The authors declare that the research was conducted in the absence of any commercial or financial relationships that could be construed as a potential conflict of interest.

## Publisher’s Note

All claims expressed in this article are solely those of the authors and do not necessarily represent those of their affiliated organizations, or those of the publisher, the editors and the reviewers. Any product that may be evaluated in this article, or claim that may be made by its manufacturer, is not guaranteed or endorsed by the publisher.

## Abbreviations

BMI, Body Mass Index; RIA, Radioimmunoassay; ELISA, Enzyme linked immunosorbent assay; USA, United States of America; UK, United Kingdom; T1DM, Type-1diabetes mellitus.
