# Supplementary material for: Chemokines in Type 1 Diabetes Mellitus
Source: Front Immunol. 2022 Feb 15;12:690082. doi: 10.3389/fimmu.2021.690082 (PMC8886728; doi:10.3389/fimmu.2021.690082)
Supplement: Supplementary file 1 [file DataSheet_1.doc]

**Supplementary appendix**

**Contents**

**Appendix 1**: Electronic search strategies

**Appendix 2**: Study selection flow chart. A flow chart demonstrating the selection process of articles included in the analysis as well as in the qualitative summary.

**Appendix 3**: Anthropometric and clinical phenomics characteristics of included studies

**Appendix 4:** The classification of chemokines and their receptors.

**Appendix 5**: The distribution—cell type of chemokines receptors.

**Appendix 6:** Egger funnel plots of T1DM patients compared to controls.

**Appendices References**

**Appendix 1**: Electronic search strategies

| **Search** | **Query** |
| --- | --- |
| 1 | chemokine* |
| 2 | ccl1 or ccl2 or ccl3 or ccl4 or ccl5 or ccl6 or ccl7 or ccl8 or ccl9 or ccl10 or ccl11 or ccl12 or ccl13 or ccl14 or ccl15 or ccl16 or ccl17 or ccl18 or ccl19 or ccl20 or ccl21 or ccl22 or ccl23 or ccl24 or ccl25 or ccl26 or ccl27 or ccl28 |
| 3 | cxcl1 or cxcl2 or cxcl3 or cxcl4 or cxcl5 or cxcl6 or cxcl7 or cxcl8 or cxcl9 or cxcl10 or cxcl11 or cxcl12 or cxcl13 or cxcl14 or cxcl15 or cxcl16 or cxcl17 |
| 4 | xcl1 or xcl2 |
| 5 | cx3cl1 |
| 6 | ccl or cxcl or xcl or cx3cl |
| 7 | scya1 or scya2 or scya3 or scya4 or scya5 or scya6 or scya7 or scya8 or scya9 or scya10 or scya11 or scya12 or scya13 or scya14 or scya15 or scya16 or scya17 or scya18 or scya19 or scya20 or scya21 or scya22 or scya23 or scya24 or scya25 or scya26 or scya27 or scya28 |
| 8 | scyb1 or scyb2 or scyb3 or scyb4 or scyb5 or scyb6 or scyb7 or scyb8 or scyb9 or scyb10 or scyb11 or scyb12 or scyb13 or scyb14 or scyb15 or scyb16 or scyb17 |
| 9 | scyc1 or scyc2 |
| 10 | sycd1 |
| 11 | scya or scyb or scyc or scyd |
| 12 | chemokine receptor* |
| 13 | ccr1 or ccr2 or ccr2b or ccr3 or ccr4 or ccr5 or ccr6 or ccr7 or ccr8 or ccr9 or ccr10 |
| 14 | cxcr1 or cxcr2 or cxcr3 or cxcr3b or cxcr4 or cxcr5 or cxcr6 or cxcr7 |
| 15 | xcr1 |
| 16 | cx3cr1 |
| 17 | ccr or cxcr or xcr or cx3cr |
| 18 | chemotactic cytokine* or chemokine* |
| 19 | i-309 or i309 or tca-3 or tca3 or sise |
| 20 | IL-8 or GCP-2 or CXCR1 or NAP-2 or ENA-78 or GROα or GROβ or GROγ or PF4 or IP-10 or MIG or I-TAC or SDF-1 or BCA-1 or SR-PSOX or BRAK or MCP-1 or MCP-4 or CCR2 or MCP-3 or MCP-2 or MIP-1β or MIP-1α or CCR5 or RANTES or MPIF-1 or HCC-1 or HCC-2 or HCC-4 or Eotaxin or Eotaxin-2 or Eotaxin-3 or TARC or CCR4 or MDC or MIP-3α or ELC or CCR7 or SLC or I-309 or TECK or CTACK or MEC or PARC or Lymphotactin or XCR1 or SCM-1β or Fractalkine or Chemerin |
| 21 | S1 OR S2 OR S3 OR S4 OR S5 OR S6 OR S7 OR S8 OR S9 OR S10 OR S11 OR S12 OR S13 OR S14 OR S15 OR S16 OR S17 OR S18 OR S19 OR S20 |
| 22 | T1DM OR Diabetes Mellitus, Type 1 OR Diabetes Mellitus, Insulin-Dependent OR Diabetes Mellitus, Insulin Dependent OR Insulin-Dependent Diabetes Mellitus |
| 23 | Diabetes Mellitus, Juvenile-Onset OR Diabetes Mellitus, Juvenile Onset OR Juvenile-Onset Diabetes Mellitus OR IDDM OR Juvenile-Onset Diabetes OR Diabetes, Juvenile-Onset OR Juvenile Onset Diabetes OR Diabetes Mellitus, Sudden-Onset OR Diabetes Mellitus, Sudden Onset OR Sudden-Onset Diabetes Mellitus OR Type 1 Diabetes Mellitus OR Diabetes Mellitus, Insulin-Dependent, 1 OR Insulin-Dependent Diabetes Mellitus 1 OR Insulin Dependent Diabetes Mellitus 1 OR Type 1 Diabetes OR Diabetes, Type 1 OR Diabetes Mellitus, Type I OR Diabetes, Autoimmune OR Autoimmune Diabetes OR Diabetes Mellitus, Brittle OR Brittle Diabetes Mellitus OR Diabetes Mellitus, Ketosis-Prone OR Diabetes Mellitus, Ketosis Prone OR Ketosis-Prone Diabetes Mellitus |
| 24 | S22 OR S23 |
| 25 | S21 AND S24 |

**768of Web of Science**

(‎TITLE: (‎(‎(‎(‎(‎(‎(‎(‎(‎(‎(‎(‎(‎(‎(‎(‎(‎(‎(‎(‎(‎(‎(‎(‎(‎(‎(‎(‎T1DM OR Diabetes Mellitus, Type 1) OR Diabetes Mellitus, Insulin-Dependent) OR Diabetes Mellitus, Insulin Dependent) OR Insulin-Dependent Diabetes Mellitus) OR Diabetes Mellitus, Juvenile-Onset) OR Diabetes Mellitus, Juvenile Onset) OR Juvenile-Onset Diabetes Mellitus) OR IDDM) OR Juvenile-Onset Diabetes) OR Diabetes, Juvenile-Onset) OR Juvenile Onset Diabetes) OR Diabetes Mellitus, Sudden-Onset) OR Diabetes Mellitus, Sudden Onset) OR Sudden-Onset Diabetes Mellitus) OR Type 1 Diabetes Mellitus) OR Diabetes Mellitus, Insulin-Dependent, 1) OR Insulin-Dependent Diabetes Mellitus 1) OR Insulin Dependent Diabetes Mellitus 1) OR Type 1 Diabetes) OR Diabetes, Type 1) OR Diabetes Mellitus, Type I) OR Diabetes, Autoimmune) OR Autoimmune Diabetes) OR Diabetes Mellitus, Brittle) OR Brittle Diabetes Mellitus) OR Diabetes Mellitus, Ketosis-Prone) OR Diabetes Mellitus, Ketosis Prone) OR Ketosis-Prone Diabetes Mellitus) AND TOPIC: (‎(‎(‎(‎(‎(‎(‎(‎(‎(‎(‎(‎(‎(‎(‎(‎(‎(‎(‎(‎(‎(‎(‎(‎(‎(‎(‎(‎(‎(‎(‎(‎(‎(‎(‎(‎(‎(‎(‎(‎(‎(‎(‎(‎(‎(‎(‎(‎(‎(‎(‎(‎(‎(‎(‎(‎(‎(‎(‎(‎(‎(‎(‎(‎(‎(‎(‎(‎(‎(‎(‎(‎(‎(‎(‎(‎(‎(‎(‎(‎(‎(‎(‎(‎(‎(‎(‎(‎(‎(‎(‎(‎(‎(‎(‎(‎(‎(‎(‎(‎(‎(‎(‎(‎(‎(‎(‎(‎(‎(‎(‎(‎(‎(‎(‎(‎(‎(‎(‎(‎(‎(‎(‎(‎(‎(‎(‎(‎(‎(‎(‎(‎(‎(‎(‎(‎(‎(‎(‎(‎(‎(‎(‎(‎(‎(‎(‎(‎(‎(‎(‎(‎(‎(‎(‎(‎(‎(‎(‎(‎(‎(‎(‎(‎(‎(‎(‎(‎(‎(‎(‎(‎(‎(‎(‎(‎(‎(‎(‎(‎(‎(‎(‎(‎(‎(‎(‎(‎(‎(‎(‎(‎(‎(‎(‎(‎(‎(‎(‎(‎(‎(‎(‎(‎(‎(‎(‎(‎(‎(‎(‎(‎(‎(‎(‎(‎(‎chemokine* OR ccl1) OR ccl2) OR ccl3) OR ccl4) OR ccl5) OR ccl4) OR ccl7) OR ccl8) OR ccl4) OR cxcl10) OR ccl21) OR cclf2) OR cxcl13) OR ccl19) OR ccl17) OR ccl19) OR ccl17) OR ccl18) OR ccl19) OR ccl20) OR ccl21) OR ccl22) OR ccl23) OR ccl2f) OR ccl25) OR ccl26) OR ccl2f) OR ccl28) OR cxcl1) OR cxcl2) OR cxcl3) OR cxcr4) OR cxcl5) OR c2cl6) OR cxcl1) OR cxcl8) OR cxcl9) OR cxcl10) OR cxcl11) OR cxcl12) OR cxcl13) OR cxcl10) OR cxcl12) OR cxcl16) OR cxcl10) OR xclx) OR xch2) OR cx3cl1) OR ccl) OR cxcl) OR xcl) OR ch3cl) OR scca1) OR scca2) OR scya2) OR scya2) OR scya2) OR scya2) OR scya2) OR scya2) OR scya2) OR scya17) OR scya17) OR scya17) OR scya17) OR scya17) OR scya17) OR scya17) OR scya11) OR scya17) OR scya11) OR scna20) OR scya11) OR scya20) OR scya20) OR scya20) OR scya20) OR scya20) OR scya17) OR scya20) OR scyl1) OR scya2) OR scya3) OR scya4) OR scya5) OR scyb5) OR scya7) OR scyb5) OR sc5b9) OR scyb11) OR scya11) OR scyb10) OR scyb10) OR scyb10) OR scyb10) OR scyb10) OR scya17) OR scyl1) OR sc2c2) OR sycp1) OR soya) OR scab) OR sclc) OR scid) OR chemokine receptor*) OR ccr1) OR ccr2) OR chr2b) OR ccr3) OR ccr4) OR ccr5) OR ccr6) OR ccr7) OR ccr8) OR ccr9) OR cor10) OR cxcr1) OR cxcr2) OR cxcr3) OR cxcr4b) OR cxcr4) OR cxcr5) OR cxcr4) OR cxcr4) OR xcr1) OR cx3cr1) OR ccr) OR cxcr) OR xcr) OR cx3cr1) OR chemotactic cytokine*) OR chemokine*) OR i-309) OR i300) OR tca-3) OR tcam) OR side) OR IL-8) OR GCP-2) OR CXCR1) OR NAP-2) OR ENA-78) OR GROα) OR GROβ) OR GROγ) OR PF4) OR IP-10) OR MIG) OR I-TAC) OR SDF-1) OR BCA-1) OR SR-PSOX) OR BRAK) OR MCP-1) OR MCP-4) OR CCR2) OR MCP-3) OR MCP-2) OR MIP-1β) OR MIP-1α) OR CCR5) OR RANTES) OR MPIF-1) OR HCC-1) OR HCC-2) OR HCC-4) OR Eotaxin) OR Eotaxin-2) OR Eotaxin-3) OR TARC) OR CCR4) OR MDC) OR MIP-3α) OR ELC) OR CCR7) OR SLC) OR I-309) OR TECK) OR CTACK) OR MEC) OR PARC) OR Lymphotactin) OR XCR1) OR SCM-1β) OR Fractalkine) OR Chemerin) OR Limphotactin) OR SCM-1) OR C-10) OR Mrp-1) OR MIP-1γ) OR MRP2) OR Eotaxin-1) OR MCP-5) OR Leukotactin-1) OR MIP-5) OR LEC) OR NCC-4) OR MTN1) OR MIP-4) OR AMAC1) OR ELC) OR MIP-3β) OR LARC) OR MIP-3) OR 6Ckine) OR MPIF-2) OR Eotaxin-2) OR MIP-4α) OR CTAK) OR MGSA) OR MIP-2α) OR MIP-2β) OR PF-4) OR BLC) OR Lungkine) OR SRPSOX))

**1228 of Embase**

(t1dm:ti,ab,kw OR 'diabetes mellitus, type 1':ti,ab,kw OR 'diabetes mellitus, insulin-dependent':ti,ab,kw OR 'diabetes mellitus, insulin dependent':ti,ab,kw OR 'insulin-dependent diabetes mellitus':ti,ab,kw OR 'diabetes mellitus, juvenile-onset':ti,ab,kw OR 'diabetes mellitus, juvenile onset':ti,ab,kw OR 'juvenile-onset diabetes mellitus':ti,ab,kw OR iddm:ti,ab,kw OR 'juvenile-onset diabetes':ti,ab,kw OR 'diabetes, juvenile-onset':ti,ab,kw OR 'juvenile onset diabetes':ti,ab,kw OR 'diabetes mellitus, sudden-onset':ti,ab,kw OR 'diabetes mellitus, sudden onset':ti,ab,kw OR 'sudden-onset diabetes mellitus':ti,ab,kw OR 'type 1 diabetes mellitus':ti,ab,kw OR 'diabetes mellitus, insulin-dependent, 1':ti,ab,kw OR 'insulin-dependent diabetes mellitus 1':ti,ab,kw OR 'insulin dependent diabetes mellitus 1':ti,ab,kw OR 'type 1 diabetes':ti,ab,kw OR 'diabetes, type 1':ti,ab,kw OR 'diabetes mellitus, type i':ti,ab,kw OR 'diabetes, autoimmune':ti,ab,kw OR 'autoimmune diabetes':ti,ab,kw OR 'diabetes mellitus, brittle':ti,ab,kw OR 'brittle diabetes mellitus':ti,ab,kw OR 'diabetes mellitus, ketosis-prone':ti,ab,kw OR 'diabetes mellitus, ketosis prone':ti,ab,kw OR 'ketosis-prone diabetes mellitus':ti,ab,kw) AND (ccl1:ti,ab,kw OR ccl2:ti,ab,kw OR ccl3:ti,ab,kw OR ccl4:ti,ab,kw OR ccl5:ti,ab,kw OR ccl6:ti,ab,kw OR ccl7:ti,ab,kw OR ccl8:ti,ab,kw OR ccl9:ti,ab,kw OR ccl10:ti,ab,kw OR ccl11:ti,ab,kw OR ccl12:ti,ab,kw OR ccl13:ti,ab,kw OR ccl14:ti,ab,kw OR ccl15:ti,ab,kw OR ccl16:ti,ab,kw OR ccl17:ti,ab,kw OR ccl18:ti,ab,kw OR ccl19:ti,ab,kw OR ccl20:ti,ab,kw OR ccl21:ti,ab,kw OR ccl22:ti,ab,kw OR ccl23:ti,ab,kw OR ccl24:ti,ab,kw OR ccl25:ti,ab,kw OR ccl26:ti,ab,kw OR ccl27:ti,ab,kw OR ccl28:ti,ab,kw OR cxcl1:ti,ab,kw OR cxcl2:ti,ab,kw OR cxcl3:ti,ab,kw OR cxcl4:ti,ab,kw OR cxcl5:ti,ab,kw OR cxcl6:ti,ab,kw OR cxcl7:ti,ab,kw OR cxcl8:ti,ab,kw OR cxcl9:ti,ab,kw OR cxcl10:ti,ab,kw OR cxcl11:ti,ab,kw OR cxcl12:ti,ab,kw OR cxcl13:ti,ab,kw OR cxcl14:ti,ab,kw OR cxcl15:ti,ab,kw OR cxcl16:ti,ab,kw OR cxcl17:ti,ab,kw OR xcl1:ti,ab,kw OR xcl2:ti,ab,kw OR cx3cl1:ti,ab,kw OR ccl:ti,ab,kw OR cxcl:ti,ab,kw OR xcl:ti,ab,kw OR cx3cl:ti,ab,kw OR scya1:ti,ab,kw OR scya2:ti,ab,kw OR scya3:ti,ab,kw OR scya4:ti,ab,kw OR scya5:ti,ab,kw OR scya6:ti,ab,kw OR scya7:ti,ab,kw OR scya8:ti,ab,kw OR scya9:ti,ab,kw OR scya10:ti,ab,kw OR scya11:ti,ab,kw OR scya12:ti,ab,kw OR scya13:ti,ab,kw OR scya14:ti,ab,kw OR scya15:ti,ab,kw OR scya16:ti,ab,kw OR scya17:ti,ab,kw OR scya18:ti,ab,kw OR scya19:ti,ab,kw OR scya20:ti,ab,kw OR scya21:ti,ab,kw OR scya22:ti,ab,kw OR scya23:ti,ab,kw OR scya24:ti,ab,kw OR scya25:ti,ab,kw OR scya26:ti,ab,kw OR scya27:ti,ab,kw OR scya28:ti,ab,kw OR scyb1:ti,ab,kw OR scyb2:ti,ab,kw OR scyb3:ti,ab,kw OR scyb4:ti,ab,kw OR scyb5:ti,ab,kw OR scyb6:ti,ab,kw OR scyb7:ti,ab,kw OR scyb8:ti,ab,kw OR scyb9:ti,ab,kw OR scyb10:ti,ab,kw OR scyb11:ti,ab,kw OR scyb12:ti,ab,kw OR scyb13:ti,ab,kw OR scyb14:ti,ab,kw OR scyb15:ti,ab,kw OR scyb16:ti,ab,kw OR scyb17:ti,ab,kw OR scyc1:ti,ab,kw OR scyc2:ti,ab,kw OR sycd1:ti,ab,kw OR scya:ti,ab,kw OR scyb:ti,ab,kw OR scyc:ti,ab,kw OR scyd:ti,ab,kw OR 'chemokine receptor*':ti,ab,kw OR ccr1:ti,ab,kw OR ccr2b:ti,ab,kw OR ccr3:ti,ab,kw OR ccr6:ti,ab,kw OR ccr8:ti,ab,kw OR ccr9:ti,ab,kw OR ccr10:ti,ab,kw OR cxcr2:ti,ab,kw OR cxcr3:ti,ab,kw OR cxcr3b:ti,ab,kw OR cxcr4:ti,ab,kw OR cxcr5:ti,ab,kw OR cxcr6:ti,ab,kw OR cxcr7:ti,ab,kw OR cx3cr1:ti,ab,kw OR ccr:ti,ab,kw OR cxcr:ti,ab,kw OR xcr:ti,ab,kw OR cx3cr:ti,ab,kw OR 'chemotactic cytokine*':ti,ab,kw OR chemokine*:ti,ab,kw OR i309:ti,ab,kw OR 'tca 3':ti,ab,kw OR tca3:ti,ab,kw OR sise:ti,ab,kw OR 'il 8':ti,ab,kw OR 'gcp 2':ti,ab,kw OR cxcr1:ti,ab,kw OR 'nap 2':ti,ab,kw OR 'ena 78':ti,ab,kw OR groα:ti,ab,kw OR groβ:ti,ab,kw OR groγ:ti,ab,kw OR pf4:ti,ab,kw OR 'ip 10':ti,ab,kw OR mig:ti,ab,kw OR 'i tac':ti,ab,kw OR 'sdf 1':ti,ab,kw OR 'bca 1':ti,ab,kw OR 'sr psox':ti,ab,kw OR brak:ti,ab,kw OR 'mcp 1':ti,ab,kw OR 'mcp 4':ti,ab,kw OR ccr2:ti,ab,kw OR 'mcp 3':ti,ab,kw OR 'mcp 2':ti,ab,kw OR 'mip 1β':ti,ab,kw OR 'mip 1α':ti,ab,kw OR ccr5:ti,ab,kw OR rantes:ti,ab,kw OR 'mpif 1':ti,ab,kw OR 'hcc 1':ti,ab,kw OR 'hcc 2':ti,ab,kw OR 'hcc 4':ti,ab,kw OR eotaxin:ti,ab,kw OR 'eotaxin 3':ti,ab,kw OR tarc:ti,ab,kw OR ccr4:ti,ab,kw OR mdc:ti,ab,kw OR 'mip 3α':ti,ab,kw OR ccr7:ti,ab,kw OR slc:ti,ab,kw OR 'i 309':ti,ab,kw OR teck:ti,ab,kw OR ctack:ti,ab,kw OR mec:ti,ab,kw OR parc:ti,ab,kw OR lymphotactin:ti,ab,kw OR xcr1:ti,ab,kw OR 'scm 1β':ti,ab,kw OR fractalkine:ti,ab,kw OR chemerin:ti,ab,kw OR limphotactin:ti,ab,kw OR 'scm 1':ti,ab,kw OR 'c 10':ti,ab,kw OR 'mrp 1':ti,ab,kw OR 'mip 1γ':ti,ab,kw OR mrp2:ti,ab,kw OR 'eotaxin 1':ti,ab,kw OR 'mcp 5':ti,ab,kw OR 'leukotactin 1':ti,ab,kw OR 'mip 5':ti,ab,kw OR lec:ti,ab,kw OR 'ncc 4':ti,ab,kw OR mtn1:ti,ab,kw OR 'mip 4':ti,ab,kw OR amac1:ti,ab,kw OR elc:ti,ab,kw OR 'mip 3β':ti,ab,kw OR larc:ti,ab,kw OR 'mip 3':ti,ab,kw OR 6ckine:ti,ab,kw OR 'mpif 2':ti,ab,kw OR 'eotaxin 2':ti,ab,kw OR 'mip 4α':ti,ab,kw OR ctak:ti,ab,kw OR mgsa:ti,ab,kw OR 'mip 2α':ti,ab,kw OR 'mip 2β':ti,ab,kw OR 'pf 4':ti,ab,kw OR blc:ti,ab,kw OR lungkine:ti,ab,kw OR srpsox:ti,ab,kw)

**903 of Pubmed**

(T1DM[Title/Abstract] OR Diabetes Mellitus, Type 1 [Title/Abstract] OR Diabetes Mellitus, Insulin-Dependent[Title/Abstract] OR Diabetes Mellitus, Insulin Dependent[Title/Abstract] OR Insulin-Dependent Diabetes Mellitus[Title/Abstract] OR Diabetes Mellitus, Juvenile-Onset[Title/Abstract] OR Diabetes Mellitus, Juvenile Onset[Title/Abstract] OR Juvenile-Onset Diabetes Mellitus[Title/Abstract] OR IDDM[Title/Abstract] OR Juvenile-Onset Diabetes[Title/Abstract] OR Diabetes, Juvenile-Onset[Title/Abstract] OR Juvenile Onset Diabetes[Title/Abstract] OR Diabetes Mellitus, Sudden-Onset[Title/Abstract] OR Diabetes Mellitus, Sudden Onset[Title/Abstract] OR Sudden-Onset Diabetes Mellitus[Title/Abstract] OR Type 1 Diabetes Mellitus[Title/Abstract] OR Diabetes Mellitus, Insulin-Dependent, 1[Title/Abstract] OR Insulin-Dependent Diabetes Mellitus 1[Title/Abstract] OR Insulin Dependent Diabetes Mellitus 1[Title/Abstract] OR Type 1 Diabetes[Title/Abstract] OR Diabetes, Type 1[Title/Abstract] OR Diabetes Mellitus, Type I[Title/Abstract] OR Diabetes, Autoimmune[Title/Abstract] OR Autoimmune Diabetes[Title/Abstract] OR Diabetes Mellitus, Brittle[Title/Abstract] OR Brittle Diabetes Mellitus[Title/Abstract] OR Diabetes Mellitus, Ketosis-Prone[Title/Abstract] OR Diabetes Mellitus, Ketosis Prone[Title/Abstract] OR Ketosis-Prone Diabetes Mellitus[Title/Abstract]) AND (chemokine*[Title/Abstract] OR ccl1[Title/Abstract] OR ccl2[Title/Abstract] OR ccl3[Title/Abstract] OR ccl4[Title/Abstract] OR ccl5[Title/Abstract] OR ccl6[Title/Abstract] OR ccl7[Title/Abstract] OR ccl8[Title/Abstract] OR ccl9[Title/Abstract] OR ccl10[Title/Abstract] OR ccl11[Title/Abstract] OR ccl12[Title/Abstract] OR ccl13[Title/Abstract] OR ccl14[Title/Abstract] OR ccl15[Title/Abstract] OR ccl16[Title/Abstract] OR ccl17[Title/Abstract] OR ccl18[Title/Abstract] OR ccl19[Title/Abstract] OR ccl20[Title/Abstract] OR ccl21[Title/Abstract] OR ccl22[Title/Abstract] OR ccl23[Title/Abstract] OR ccl24[Title/Abstract] OR ccl25[Title/Abstract] OR ccl26[Title/Abstract] OR ccl27[Title/Abstract] OR ccl28[Title/Abstract] OR cxcl1[Title/Abstract] OR cxcl2[Title/Abstract] OR cxcl3[Title/Abstract] OR cxcl4[Title/Abstract] OR cxcl5[Title/Abstract] OR cxcl6[Title/Abstract] OR cxcl7[Title/Abstract] OR cxcl8[Title/Abstract] OR cxcl9[Title/Abstract] OR cxcl10[Title/Abstract] OR cxcl11[Title/Abstract] OR cxcl12[Title/Abstract] OR cxcl13[Title/Abstract] OR cxcl14[Title/Abstract] OR cxcl15[Title/Abstract] OR cxcl16[Title/Abstract] OR cxcl17[Title/Abstract] OR xcl1[Title/Abstract] OR xcl2[Title/Abstract] OR cx3cl1[Title/Abstract] OR ccl[Title/Abstract] OR cxcl[Title/Abstract] OR xcl[Title/Abstract] OR cx3cl[Title/Abstract] OR scya1[Title/Abstract] OR scya2[Title/Abstract] OR scya3[Title/Abstract] OR scya4[Title/Abstract] OR scya5[Title/Abstract] OR scya6[Title/Abstract] OR scya7[Title/Abstract] OR scya8[Title/Abstract] OR scya9[Title/Abstract] OR scya10[Title/Abstract] OR scya11[Title/Abstract] OR scya12[Title/Abstract] OR scya13[Title/Abstract] OR scya14[Title/Abstract] OR scya15[Title/Abstract] OR scya16[Title/Abstract] OR scya17[Title/Abstract] OR scya18[Title/Abstract] OR scya19[Title/Abstract] OR scya20[Title/Abstract] OR scya21[Title/Abstract] OR scya22[Title/Abstract] OR scya23[Title/Abstract] OR scya24[Title/Abstract] OR scya25[Title/Abstract] OR scya26[Title/Abstract] OR scya27[Title/Abstract] OR scya28[Title/Abstract] OR scyb1[Title/Abstract] OR scyb2[Title/Abstract] OR scyb3[Title/Abstract] OR scyb4[Title/Abstract] OR scyb5[Title/Abstract] OR scyb6[Title/Abstract] OR scyb7[Title/Abstract] OR scyb8[Title/Abstract] OR scyb9[Title/Abstract] OR scyb10[Title/Abstract] OR scyb11[Title/Abstract] OR scyb12[Title/Abstract] OR scyb13[Title/Abstract] OR scyb14[Title/Abstract] OR scyb15[Title/Abstract] OR scyb16[Title/Abstract] OR scyb17[Title/Abstract] OR scyc1[Title/Abstract] OR scyc2[Title/Abstract] OR sycd1[Title/Abstract] OR scya[Title/Abstract] OR scyb[Title/Abstract] OR scyc[Title/Abstract] OR scyd[Title/Abstract] OR chemokine receptor*[Title/Abstract] OR ccr1[Title/Abstract] OR ccr2[Title/Abstract] OR ccr2b[Title/Abstract] OR ccr3[Title/Abstract] OR ccr4[Title/Abstract] OR ccr5[Title/Abstract] OR ccr6[Title/Abstract] OR ccr7[Title/Abstract] OR ccr8[Title/Abstract] OR ccr9[Title/Abstract] OR ccr10[Title/Abstract] OR cxcr1[Title/Abstract] OR cxcr2[Title/Abstract] OR cxcr3[Title/Abstract] OR cxcr3b[Title/Abstract] OR cxcr4[Title/Abstract] OR cxcr5[Title/Abstract] OR cxcr6[Title/Abstract] OR cxcr7[Title/Abstract] OR xcr1[Title/Abstract] OR cx3cr1[Title/Abstract] OR ccr[Title/Abstract] OR cxcr[Title/Abstract] OR xcr[Title/Abstract] OR cx3cr[Title/Abstract] OR chemotactic cytokine*[Title/Abstract] OR chemokine*[Title/Abstract] OR i-309[Title/Abstract] OR i309[Title/Abstract] OR tca-3[Title/Abstract] OR tca3[Title/Abstract] OR sise[Title/Abstract] OR IL-8[Title/Abstract] OR GCP-2[Title/Abstract] OR CXCR1[Title/Abstract] OR NAP-2[Title/Abstract] OR ENA-78[Title/Abstract] OR GROα[Title/Abstract] OR GROβ[Title/Abstract] OR GROγ[Title/Abstract] OR PF4[Title/Abstract] OR IP-10[Title/Abstract] OR MIG[Title/Abstract] OR I-TAC[Title/Abstract] OR SDF-1[Title/Abstract] OR BCA-1[Title/Abstract] OR SR-PSOX[Title/Abstract] OR BRAK[Title/Abstract] OR MCP-1[Title/Abstract] OR MCP-4[Title/Abstract] OR CCR2[Title/Abstract] OR MCP-3[Title/Abstract] OR MCP-2[Title/Abstract] OR MIP-1β[Title/Abstract] OR MIP-1α[Title/Abstract] OR CCR5[Title/Abstract] OR RANTES[Title/Abstract] OR MPIF-1[Title/Abstract] OR HCC-1[Title/Abstract] OR HCC-2[Title/Abstract] OR HCC-4[Title/Abstract] OR Eotaxin[Title/Abstract] OR Eotaxin-2[Title/Abstract] OR Eotaxin-3[Title/Abstract] OR TARC[Title/Abstract] OR CCR4[Title/Abstract] OR MDC[Title/Abstract] OR MIP-3α[Title/Abstract] OR ELC[Title/Abstract] OR CCR7[Title/Abstract] OR SLC[Title/Abstract] OR I-309[Title/Abstract] OR TECK[Title/Abstract] OR CTACK[Title/Abstract] OR MEC[Title/Abstract] OR PARC[Title/Abstract] OR Lymphotactin[Title/Abstract] OR XCR1[Title/Abstract] OR SCM-1β[Title/Abstract] OR Fractalkine[Title/Abstract] OR Chemerin[Title/Abstract] OR Limphotactin[Title/Abstract] OR SCM-1[Title/Abstract] OR C-10[Title/Abstract] OR Mrp-1[Title/Abstract] OR MIP-1γ[Title/Abstract] OR MRP2[Title/Abstract] OR Eotaxin-1[Title/Abstract] OR MCP-5[Title/Abstract] OR Leukotactin-1[Title/Abstract] OR MIP-5[Title/Abstract] OR LEC[Title/Abstract] OR NCC-4[Title/Abstract] OR MTN1[Title/Abstract] OR MIP-4[Title/Abstract] OR AMAC1[Title/Abstract] OR ELC[Title/Abstract] OR MIP-3β[Title/Abstract] OR LARC[Title/Abstract] OR MIP-3[Title/Abstract] OR 6Ckine[Title/Abstract] OR MPIF-2[Title/Abstract] OR Eotaxin-2[Title/Abstract] OR MIP-4α[Title/Abstract] OR CTAK[Title/Abstract] OR MGSA[Title/Abstract] OR MIP-2α[Title/Abstract] OR MIP-2β[Title/Abstract] OR PF-4[Title/Abstract] OR BLC[Title/Abstract] OR Lungkine[Title/Abstract] OR SRPSOX[Title/Abstract])

**833 of Cochrane Library**

chemokine* or ccl1 or ccl2 or ccl3 or ccl4 or ccl5 or ccl6 or ccl7 or ccl8 or ccl9 or ccl10 or ccl11 or ccl12 or ccl13 or ccl14 or ccl15 or ccl16 or ccl17 or ccl18 or ccl19 or ccl20 or ccl21 or ccl22 or ccl23 or ccl24 or ccl25 or ccl26 or ccl27 or ccl28 or cxcl1 or cxcl2 or cxcl3 or cxcl4 or cxcl5 or cxcl6 or cxcl7 or cxcl8 or cxcl9 or cxcl10 or cxcl11 or cxcl12 or cxcl13 or cxcl14 or cxcl15 or cxcl16 or cxcl17 or xcl1 or xcl2 or cx3cl1 or ccl or cxcl or xcl or cx3cl or scya1 or scya2 or scya3 or scya4 or scya5 or scya6 or scya7 or scya8 or scya9 or scya10 or scya11 or scya12 or scya13 or scya14 or scya15 or scya16 or scya17 or scya18 or scya19 or scya20 or scya21 or scya22 or scya23 or scya24 or scya25 or scya26 or scya27 or scya28 or scyb1 or scyb2 or scyb3 or scyb4 or scyb5 or scyb6 or scyb7 or scyb8 or scyb9 or scyb10 or scyb11 or scyb12 or scyb13 or scyb14 or scyb15 or scyb16 or scyb17 or scyc1 or scyc2 or sycd1 or scya or scyb or scyc or scyd or chemokine receptor* or ccr1 or ccr2 or ccr2b or ccr3 or ccr4 or ccr5 or ccr6 or ccr7 or ccr8 or ccr9 or ccr10 or cxcr1 or cxcr2 or cxcr3 or cxcr3b or cxcr4 or cxcr5 or cxcr6 or cxcr7 or xcr1 or cx3cr1 or ccr or cxcr or xcr or cx3cr or chemotactic cytokine* or chemokine* or i-309 or i309 or tca-3 or tca3 or sise or IL-8 or GCP-2 or CXCR1 or NAP-2 or ENA-78 or GROα or GROβ or GROγ or PF4 or IP-10 or MIG or I-TAC or SDF-1 or BCA-1 or SR-PSOX or BRAK or MCP-1 or MCP-4 or CCR2 or MCP-3 or MCP-2 or MIP-1β or MIP-1α or CCR5 or RANTES or MPIF-1 or HCC-1 or HCC-2 or HCC-4 or Eotaxin or Eotaxin-2 or Eotaxin-3 or TARC or CCR4 or MDC or MIP-3α or ELC or CCR7 or SLC or I-309 or TECK or CTACK or MEC or PARC or Lymphotactin or XCR1 or SCM-1β or Fractalkine or Chemerin or Limphotactin or SCM-1 or C-10 or Mrp-1 or MIP-1γ or MRP2 or Eotaxin-1 or MCP-5 or Leukotactin-1 or MIP-5 or LEC or NCC-4 or MTN1 or MIP-4 or AMAC1 or ELC or MIP-3β or LARC or MIP-3 or 6Ckine or MPIF-2 or Eotaxin-2 or MIP-4α or CTAK or MGSA or MIP-2α or MIP-2β or PF-4 or BLC or Lungkine or SRPSOX in Title Abstract Keyword AND T1DM OR Diabetes Mellitus, Type 1 OR Diabetes Mellitus, Insulin-Dependent OR Diabetes Mellitus, Insulin Dependent OR Insulin-Dependent Diabetes Mellitus OR Diabetes Mellitus, Juvenile-Onset OR Diabetes Mellitus, Juvenile Onset OR Juvenile-Onset Diabetes Mellitus OR IDDM OR Juvenile-Onset Diabetes OR Diabetes, Juvenile-Onset OR Juvenile Onset Diabetes OR Diabetes Mellitus, Sudden-Onset OR Diabetes Mellitus, Sudden Onset OR Sudden-Onset Diabetes Mellitus OR Type 1 Diabetes Mellitus OR Diabetes Mellitus, Insulin-Dependent, 1 OR Insulin-Dependent Diabetes Mellitus 1 OR Insulin Dependent Diabetes Mellitus 1 OR Type 1 Diabetes OR Diabetes, Type 1 OR Diabetes Mellitus, Type I OR Diabetes, Autoimmune OR Autoimmune Diabetes OR Diabetes Mellitus, Brittle OR Brittle Diabetes Mellitus OR Diabetes Mellitus, Ketosis-Prone OR Diabetes Mellitus, Ketosis Prone OR Ketosis-Prone Diabetes Mellitus in Title Abstract Keyword

**Appendix 2: Study selection flow chart. A flow chart demonstrating the selection process of articles included in the analysis as well as in the qualitative summary.**


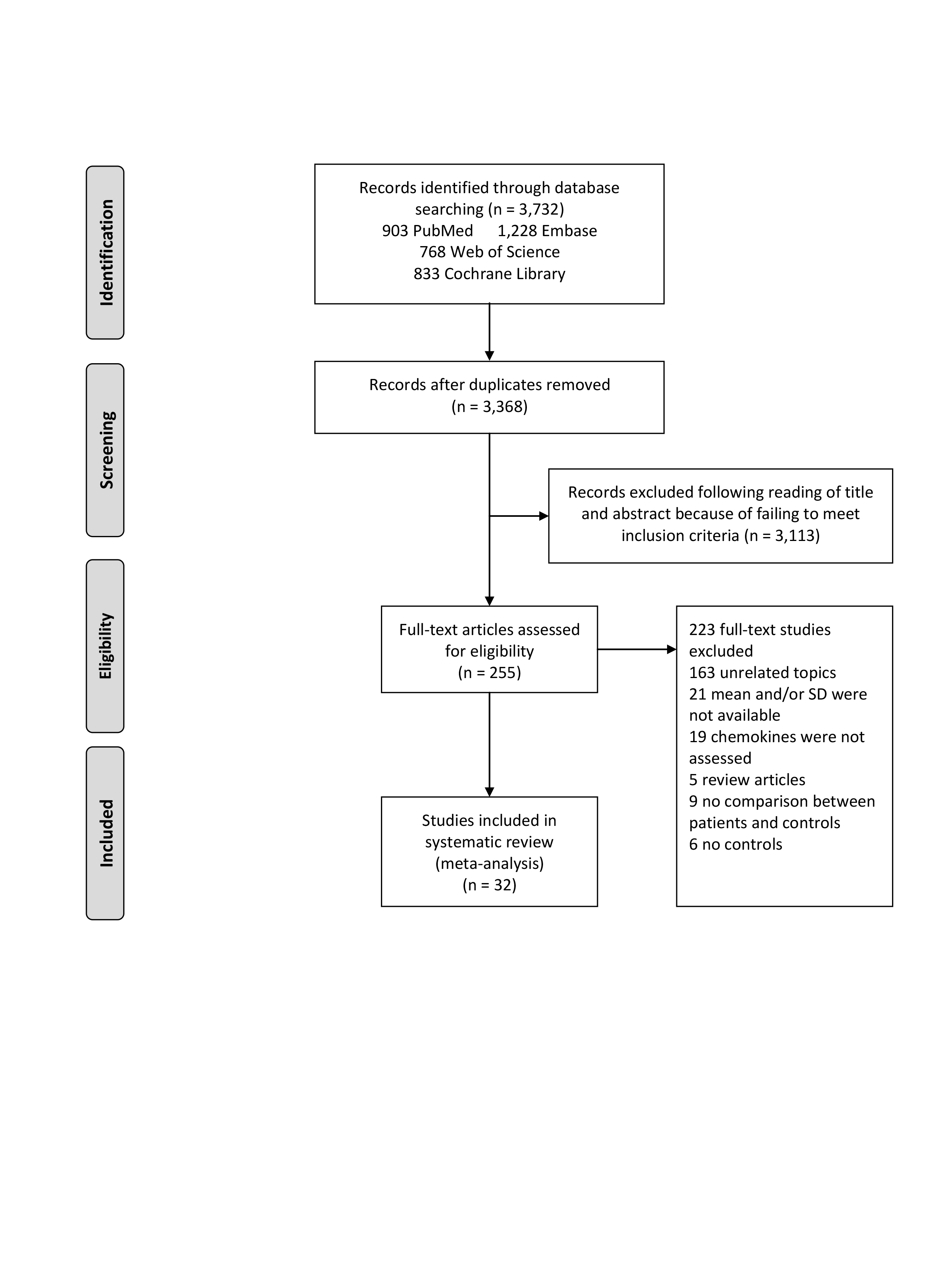


**Appendix 3: Anthropometric and clinical phenomics c**haracteristics of included studies

| **Study** |  | **Waist circumference (cm)** | **SBP (mmHg)** | **DBP (mmHg)** | **FPG (mmol/L)** | **HbA1c (%)** | **Creatinine (μmol/L)** | **Cholesterol** | **TG** | **LDL (mmol/L)** | **HDL (mmol/L)** | **CRP (mg/L)** |
| --- | --- | --- | --- | --- | --- | --- | --- | --- | --- | --- | --- | --- |
| **Abke 2006** | [1] | NR | NR | NR | NR | 7.2 (5.7-9.5) | NR | NR | NR | NR | NR | 1.1 (0.7-1.6) mg/L |
| **Antonelli 2009** | [2] | NR | NR | NR | 18.6±9.10 | 9.7±2.9 | NR | NR | NR | NR | NR | NR |
| **Berg 2010** | [3] | NR | NR | NR | NR | NR | NR | NR | NR | NR | NR | NR |
| **Cetinkalp 2015** | [4] | NR | NR | NR | NR | 6.1 (5.1-6.9) | NR | 186.8 mg/dL | 65.9 mg/dL | 102.1 mg/dL | 71.5 mg/dL | 0.3±0.6 mg/dL |
| **Chatzigeorgiou 2010** | [5] | NR | NR | NR | NR | 8.45±0.43 | 78.0±5.0 | 165.22±7.14 mg/dL | 82.08±6.58 mg/dL | 91.14±4.28 mg/dL | 58.14±1.81 mg/dL | 6.22±0.25 mg/dL |
| **Dakovic2013** | [6] | NR | NR | NR | NR | 8.79±0.84 | NR | NR | NR | NR | NR | NR |
| **Ellina 2012** | [7] | NR | NR | NR | NR | 7.67±0.81 | 62.0±4.3 | 168.59±7.23 mg/dL | 80.02±5.55 mg/dL | 89.58±10.12 mg/dL | 61.58±9.81 mg/dL | 6.54±1.45 mg/dL |
| **Erbag˘c 2001** | [8] | NR | NR | NR | NR | NR | NR | NR | NR | NR | NR | NR |
| **Gabbay 2012** | [9] | NR | NR | NR | NR | 8.4±2.2 | NR | NR | NR | NR | NR | NR |
| **Giulietti 2006** | [10] | NR | NR | NR | 142 (54-209) | 9.9 (6.7-13.6) | NR | NR | NR | NR | NR | NR |
| **Guan 2011** | [11] | NR | NR | NR | NR | NR | NR | NR | NR | NR | NR | NR |
| **Hakimizadeh 2013** | [12] | NR | NR | NR | 227.6±8.9 | 9.1±0.23 | NR | NR | NR | NR | NR | NR |
| **Heier 2015** | [13] | 71.2 (10.0) | 101.0±10.1 | 60.5±8.3 | NR | 8.4±1.2) | 70.0 | 4.6 (0.8) mmol/L | 0.7 (0.5-0.9) mmol/L | 2.5±0.7 mmol/L | 1.8±0.4 mmol/L | 0.51(0.27,1.77) mg/L |
| **Huang 2012** | [14] | NR | NR | NR | NR | NR | NR | NR | NR | NR | NR | NR |
| **Ismail 2016** | [15] | NR | 105.38±12.74 | 68.07±9.77 | NR | 8.35±2.07 | NR | 189.16±60.51 mg/dL | 104.72±50.38 mg/dL | 108.12±64.56 mg/dL | 43.94±14.36 mg/dL | NR |
| **Jamali 2013** | [16] | NR | NR | NR | 227.63±8.95 | 9.11±0.23 | NR | NR | NR | NR | NR | NR |
| **Lappin 2015** | [17] | NR | NR | NR | 11.3±2.5 | 7.6±1.3 | NR | NR | NR | NR | NR | NR |
| **Lohmann 2002** | [18] | NR | NR | NR | NR | 10.6±2.1 | NR | NR | NR | NR | NR | NR |
| **Melo 2016** | [19] | 77.7±11.8 | 116.1±12.4 | 67.8±7.0 | NR | 8.5±1.1 | 53.0±7.3 | 4.2±0.9 mmol/L | 0.84±0.4 mmol/L | 2.26±0.7 mmol/L | 1.57±0.4 mmol/L | NR |
| **Nicolett 2002** | [20] | NR | NR | NR | NR | NR | NR | NR | NR | NR | NR | NR |
| **Nieminen 2012** | [21] | NR | NR | NR | NR | NR | NR | NR | NR | NR | NR | NR |
| **Pellegrini 2017** | [22] | NR | NR | NR | NR | 8.2 (5.7-14) | NR | NR | NR | NR | NR | NR |
| **Pham 2012** | [23] | NR | 120 (110-139) | 80(68-85) | NR | NR | NR | NR | NR | NR | NR | NR |
| **Powell 2018** | [24] | NR | NR | NR | NR | NR | NR | NR | NR | NR | NR | NR |
| **Purohit 2015** | [25] | NR | NR | NR | NR | NR | NR | NR | NR | NR | NR | NR |
| **Rosa 2008** | [26] | NR | NR | NR | 5.5±0.1 | 7.5±0.05 | NR | NR | NR | NR | NR | NR |
| **Sickle 2010** | [27] | NR | NR | NR | NR | 7.5±0.6 | NR | NR | NR | NR | NR | NR |
| **Sochet 2017** | [28] | 74.5±9.4 | 115.0±9.3 | 67.4±6.7 | 9.97±4.46 | 9.0±1.1 | 53.6±9.9 | 4.2±0.8 mmol/L | 0.85±0.32 mmol/L | 2.22±0.60 mmol/L | 1.64±0.34 mmol/L | NR |
| **Vorobjova 2019** | [29] | NR | NR | NR | NR | NR | NR | NR | NR | NR | NR | NR |
| **Wolkow 2008** | [30] | NR | 127 (114-137) | 73 (69-78) | NR | 8.0 (7.2-8.7) | NR | NR | NR | NR | NR | NR |
| **Yamamura 2019** | [31] | NR | NR | NR | 150.6±50.1 | 8.1±1.7 | NR | NR | NR | NR | NR | NR |
| **Zóka 2015** | [32] | NR | NR | NR | NR | 7.3 (6.8-7.7) | NR | NR | NR | NR | NR | NR |

NR, not report; WC, waist circumference; FPG, fasting plasma glucose; SBP, systolic blood pressure; DBP, diastolic blood pressure; HbA1c, haemoglobin A1c; CRP, C-reactive protein; LDL, low-density lipoprotein; HDL, high-density lipoprotein; TG, triglycerides.

**Appendix 4**: The classification of chemokines and their receptors.

| **Subfamily of chemokine** | **Name of chemokine** | **Other names of chemokine** | **Receptors** |
| --- | --- | --- | --- |
| **C chemokine** | XCL1 | Limphotactin α, SCM-1α | XCR1 |
|  | XCL2 | Limphotactin β, SCM-1β | XCR1 |
| **CC chemokine** | CCL1 | I-309 | CCR8 |
|  | CCL2 | MCP-1 | CCR2 |
|  | CCL3 | MIP-1α | CCR1,CCR5 |
|  | CCL4 | MIP-1β | CCR5,CCR8 |
|  | CCL5 | RANTES | CCR1,CCR3,CCR5 |
|  | CCL6 | C-10,Mrp-1 | CCR1 |
|  | CCL7 | MCP-3 | CCR1,CCR2,CCR3 |
|  | CCL8 | MCP-2 | CCR1,CCR2,CCR3,CCR5,CCR8 |
|  | CCL9 | MIP-1γ,MRP2 | CCR1,CCR3 |
|  | CCL11 | Eotaxin-1 | CCR3,CCR5 |
|  | CCL12 | MCP-5 | CCR2 |
|  | CCL13 | MCP-4 | CCR1,CCR2，CCR3,CCR5 |
|  | CCL14 | HCC-1 | CCR1,CCR5 |
|  | CCL15 | HCC-2, Leukotactin-1, MIP-5 | CCR1,CCR3 |
|  | CCL16 | HCC-4, LEC, NCC-4, MTN1 | CCR1,CCR2,CCR5,CCR8 |
|  | CCL17 | TARC | CCR4 |
|  | CCL18 | MIP-4, AMAC1 | CCR8 |
|  | CCL19 | ELC,MIP-3β | CCR7 |
|  | CCL20 | LARC,MIP-3α | CCR6 |
|  | CCL21 | SLC,6Ckine | CCR7 |
|  | CCL22 | MDC | CCR4 |
|  | CCL23 | MPIF-1,MIP-3 | CCR1,CCR3 |
|  | CCL24 | MPIF-2,Eotaxin-2 | CCR3 |
|  | CCL25 | TECK | CCR9 |
|  | CCL26 | Eotaxin-3,MIP-4α | CCR3,CX3CR1 |
|  | CCL27 | CTAK | CCR10 |
|  | CCL28 | MEC | CCR3,CCR10 |
| **CXC chemokine** | CXCL1 | GROα,MGSA | CXCR2 |
|  | CXCL2 | GROβ,MIP-2α | CXCR2 |
|  | CXCL3 | GROγ,MIP-2β | CXCR2 |
|  | CXCL4 | PF-4 | CXCR3 |
|  | CXCL5 | ENA-78 | CXCR2 |
|  | CXCL6 | GCP-2 | CXCR1,CXCR2 |
|  | CXCL7 | NAP-2 | CXCR2 |
|  | CXCL8 | IL-8 | CXCR1,CXCR2 |
|  | CXCL9 | Mig | CXCR3 |
|  | CXCL10 | IP-10 | CXCR3 |
|  | CXCL11 | I-TAC | CXCR3,CXCR7 |
|  | CXCL12 | SDF-1 | CXCR4,CXCR7 |
|  | CXCL13 | BCA-1,BLC | CXCR5,CXCR3 |
|  | CXCL14 | BRAK | Unknown |
|  | CXCL15 | Lungkine | Unknown |
|  | CXCL16 | SRPSOX | CXCR6 |
| **CX3C chemokine** | CX3CL1 | Fractalkine | CX3CR1 |

**Appendix 5**: The distribution—cell type of chemokines receptors.

| **Receptors of Chemokine** | **Distribution—Cell Type** |
| --- | --- |
| **CXCR1** | Neutrophils, monocytes, mast cells, basophils, dendric cells, CD8 T cells,natural killer cells |
| **CXCR2** | Neutrophils, monocytes, mast cells, basophils, dendric cells, natural killer cells |
| **CXCR3** | Basophils, Th1 cells, CD8 T cells, natural killer cells, Treg cells |
| **CXCR4** | Widely expressed |
| **CXCR5** | Basophils, CD8 T cells |
| **CXCR6** | Th1 cells, Th17 cells, natural killer cells, plasma cells |
| **CCR2** | Monocytes, macrophages, Th1 cells, basophil, natural killer cells |
| **CCR5** | Dendric cells, monocytes, macrophages, natural killer cells, Th1 cells, TH17 cells, |
| **CCR1** | Neutrophils, monocytes, macrophages, Th1 cells, basophils, dendric cells |
| **CCR3** | Eosinophils, basophils, Th2 cells, mast cells, dendric cells |
| **CCR4** | Th2 cells, Th17 cells, Treg cells, monocytes, basophils, CD4 & CD8 T cells |
| **CCR6** | Th17 cells, natural killer cells, Treg cells |
| **CCR7** | Dendric cells (mature), T cells, basophils |
| **CCR8** | Dendirc cells, monocytes, macrophages, Th2 cells, Treg cells |
| **CCR9** | Basophils, dendric cells |
| **CCR10** | T cells, IgA+ plasma cells |
| **XCR1** | Dendric cells |
| **CX3CR1** | Monocytes, macrophages, Th1 cells, dendric cells, natural killer cells |

**Appendix 6: Egger funnel plots of T1DM patients compared to controls.**

**
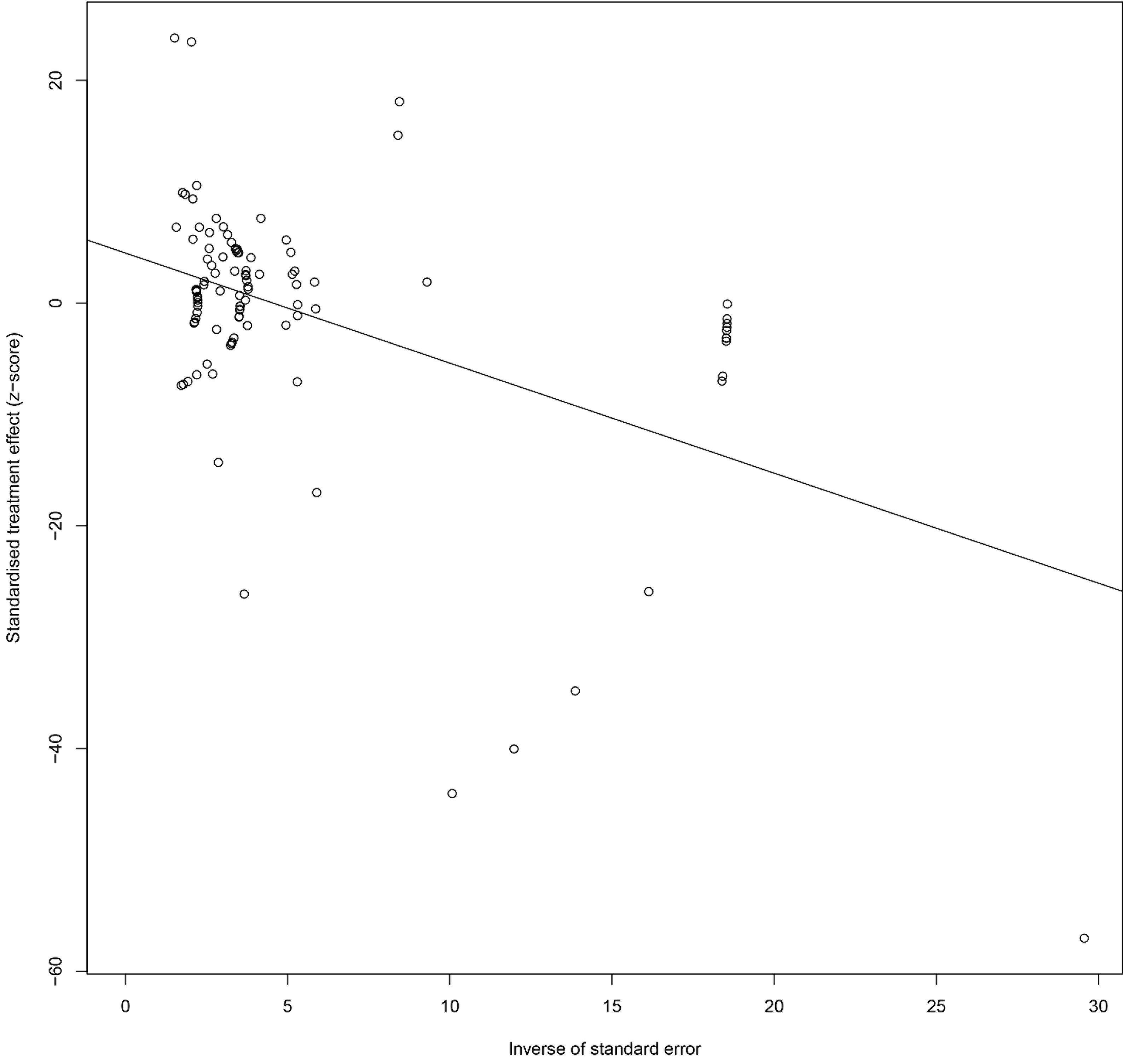
**

T1DM patients chemokine and chemokines receptor compared to patients with controls, t = 1.6357, p-value = 0.1139. Egger funnel plots to assess publication bias. Plots show study size as a function of effect size for studies included in the meta-analysis. The dots represent each study. T1DM, Type-1diabetes mellitus.

**References**

1 Abke S, Neumeier M, Weigert J, Wehrwein G, Eggenhofer E, Schäffler A, et al. Adiponectin-induced secretion of interleukin-6 (IL-6), monocyte chemotactic protein-1 (MCP-1, CCL2) and interleukin-8 (IL-8, CXCL8) is impaired in monocytes from pateints with type I diabetes. Cardiovascular Diabetology. 2006;5.

2 Antonelli A, Fallahi P, Ferrari SM, Pupilli C, d'Annunzio G, Lorini R, et al. Serum Th1 (CXCL10) and Th2 (CCL2) chemokine levels in children with newly diagnosed Type 1 diabetes: a longitudinal study. Diabetic Medicine. 2008;25(11):1349-53.

3 Berg AK, Tuvemo T, Frisk G. Enterovirus markers and serum CXCL10 in children with type 1 diabetes. Journal of Medical Virology. 2010;82(9):1594-99.

4 Cetinkalp S, Felekoglu C, Karadeniz M, Boyacioglu H, Delen Y, Yildirim E, et al. Comparison of the effects of intensive insulin treatment modalities on cardiovascular biomarkers in type 1 diabetes mellitus. Diabetes and metabolic syndrome: clinical research and reviews. 2015;9(3):157‐62.

5 Chatzigeorgiou A, Harokopos V, Mylona-Karagianni C, Tsouvalas E, Aidinis V, Kamper E. The pattern of inflammatory/anti-inflammatory cytokines and chemokines in type 1 diabetic patients over time. Annals of Medicine. 2010;42(6):426-38.

6 Dakovic D, Colic M, Cakic S, Mileusnic I, Hajdukovic Z, Stamatovic N. Salivary interleukin-8 levels in children suffering from type 1 diabetes mellitus. The Journal of clinical pediatric dentistry. 2013;37(4):377-80.

7 Ellina O, Chatzigeorgiou A, Kouyanou S, Lymberi M, Mylona-Karagianni C, Tsouvalas E, et al. Extracellular matrix-associated (GAGs, CTGF), angiogenic (VEGF) and inflammatory factors (MCP-1, CD40, IFN-gamma) in type 1 diabetes mellitus nephropathy. Clin Chem Lab Med. 2012;50(1):167-74.

8 Erbağci AB, Tarakçioğlu M, Coşkun Y, Sivasli E, Sibel Namiduru E. Mediators of inflammation in children with type I diabetes mellitus: cytokines in type I diabetic children. Clin Biochem. 2001;34(8):645-50.

9 Gabbay MA, Sato MN, Duarte AJ, Dib SA. Serum titres of anti-glutamic acid decarboxylase-65 and anti-IA-2 autoantibodies are associated with different immunoregulatory milieu in newly diagnosed type 1 diabetes patients. Clinical and experimental immunology. 2012;168(1):60-7.

10 Giulietti A, van Etten E, Overbergh L, Stoffels K, Bouillon R, Mathieu C. Monocytes from type 2 diabetic patients have a pro-inflammatory profile. 1,25-Dihydroxyvitamin D(3) works as anti-inflammatory. Diabetes Res Clin Pract. 2007;77(1):47-57.

11 Guan R, Purohit S, Wang H, Bode B, Reed JC, Steed RD, et al. Chemokine (C-C motif) ligand 2 (CCL2) in sera of patients with type 1 diabetes and diabetic complications. PLoS One. 2011;6(4):e17822.

12 Hakimizadeh E, Shamsizadeh A, Nazari M, Arababadi MK, Rezaeian M, Vazirinejad R, et al. Increased circulating levels of CXC chemokines is correlated with duration and complications of the disease in type-1 diabetes: A study on Iranian diabetic patients. Clinical Laboratory. 2013;59(5-6):531-37.

13 Heier M, Margeirsdottir HD, Brunborg C, Hanssen KF, Dahl-Jorgensen K, Seljeflot I. Inflammation in childhood type 1 diabetes; influence of glycemic control. Atherosclerosis. 2015;238(1):33-7.

14 Huang G, Mo X, Li M, Xiang Y, Li X, Luo S, et al. Autoantibodies to CCL3 are of low sensitivity and specificity for the diagnosis of type 1 diabetes. Acta Diabetologica. 2012;49(5):395-99.

15 Ismail NA, Abd El Baky AN, Ragab S, Hamed M, Hashish MA, Shehata A. Monocyte chemoattractant protein 1 and macrophage migration inhibitory factor in children with type 1 diabetes. J Pediatr Endocrinol Metab. 2016;29(6):641-5.

16 Jamali Z, Nazari M, Khoramdelazad H, Hakimizadeh E, Mahmoodi M, Karimabad MN, et al. Expression of CC chemokines CCL2, CCL5, and CCL11 is associated with duration of disease and complications in type-1 diabetes: A study on Iranian diabetic patients. Clinical Laboratory. 2013;59(9-10):993-1001.

17 Lappin DF, Robertson D, Hodge P, Treagus D, Awang RA, Ramage G, et al. The Influence of Glycated Hemoglobin on the Cross Susceptibility Between Type 1 Diabetes Mellitus and Periodontal Disease. Journal of periodontology. 2015;86(11):1249-59.

18 Lohmann T, Laue S, Nietzschmann U, Kapellen TM, Lehmann I, Schroeder S, et al. Reduced expression of Th1-associated chemokine receptors on peripheral blood lymphocytes at diagnosis of type 1 diabetes. Diabetes. 2002;51(8):2474-80.

19 De Melo EN, Deda L, Har R, Reich HN, Scholey JW, Daneman D, et al. The urinary inflammatory profile in gluten free diet-adherent adolescents with type 1 diabetes and celiac disease. Journal of diabetes and its complications. 2016;30(2):295-9.

20 Nicoletti F, Conget I, Di Mauro M, Di Marco R, Mazzarino M, Bendtzen K, et al. Serum concentrations of the interferon-γ-inducible chemokine IP-10/CXCL10 are augmented in both newly diagnosed type I diabetes mellitus patients and subjects at risk of developing the disease. Diabetologia. 2002;45(8):1107-10.

21 Nieminen JK, Vakkila J, Salo HM, Ekström N, Härkönen T, Ilonen J, et al. Altered phenotype of peripheral blood dendritic cells in pediatric type 1 diabetes. Diabetes Care. 2012;35(11):2303-10.

22 Pellegrini S, Sordi V, Bolla AM, Saita D, Ferrarese R, Canducci F, et al. Duodenal Mucosa of Patients With Type 1 Diabetes Shows Distinctive Inflammatory Profile and Microbiota. J Clin Endocrinol Metab. 2017;102(5):1468-77.

23 Pham MN, Hawa MI, Roden M, Schernthaner G, Pozzilli P, Buzzetti R, et al. Increased serum concentrations of adhesion molecules but not of chemokines in patients with Type2 diabetes compared with patients with Type1 diabetes and latent autoimmune diabetes in adult age: Action LADA5. Diabetic Medicine. 2012;29(4):470-78.

24 Powell WE, Hanna SJ, Hocter CN, Robinson E, Davies J, Dunseath GJ, et al. Loss of CXCR3 expression on memory B cells in individuals with long-standing type 1 diabetes. Diabetologia. 2018;61(8):1794-803.

25 Purohit S, Sharma A, Hopkins D, Steed L, Bode B, Anderson SW, et al. Large-scale discovery and validation studies demonstrate significant reductions in circulating levels of IL8, IL-1Ra, MCP-1, and MIP-1β in patients with type 1 diabetes. Journal of Clinical Endocrinology and Metabolism. 2015;100(9):E1179-E87.

26 Rosa JS, Oliver SR, Mitsuhashi M, Flores RL, Pontello AM, Zaldivar FP, et al. Altered kinetics of interleukin-6 and other inflammatory mediators during exercise in children with type 1 diabetes. Journal of investigative medicine : the official publication of the American Federation for Clinical Research. 2008;56(4):701-13.

27 Van Sickle BJ, Simmons J, Hall R, Raines M, Ness K, Spagnoli A. Increased circulating IL-8 is associated with reduced IGF-1 and related to poor metabolic control in adolescents with type 1 diabetes mellitus. Cytokine. 2009;48(3):290-94.

28 Sochett E, Noone D, Grattan M, Slorach C, Moineddin R, Elia Y, et al. Relationship between serum inflammatory markers and vascular function in a cohort of adolescents with type 1 diabetes. Cytokine. 2017;99:233-39.

29 Vorobjova T, Tagoma A, Oras A, Alnek K, Kisand K, Talja I, et al. Celiac Disease in Children, Particularly with Accompanying Type 1 Diabetes, Is Characterized by Substantial Changes in the Blood Cytokine Balance, Which May Reflect Inflammatory Processes in the Small Intestinal Mucosa. Journal of immunology research. 2019;2019:6179243.

30 Wolkow PP, Niewczas MA, Perkins B, Ficociello LH, Lipinski B, Warram JH, et al. Association of urinary inflammatory markers and renal decline in microalbuminuric type 1 diabetics. Journal of the American Society of Nephrology : JASN. 2008;19(4):789-97.

31 Yamamura S, Fukui T, Mori Y, Hayashi T, Yamamoto T, Ohara M, et al. Circulating anti-glutamic acid decarboxylase-65 antibody titers are positively associated with the capacity of insulin secretion in acute-onset type 1 diabetes with short duration in a Japanese population. J Diabetes Investig. 2019;10(6):1480-89.

32 Zoka A, Barna G, Hadarits O, Al-Aissa Z, Wichmann B, Muzes G, et al. Altered crosstalk in the dipeptidyl peptidase-4-incretin-immune system in type 1 diabetes: A hypothesis generating pilot study. Human immunology. 2015;76(9):667-72.
